# Articular Chondrocyte Phenotype Regulation through the Cytoskeleton and the Signaling Processes That Originate from or Converge on the Cytoskeleton: Towards a Novel Understanding of the Intersection between Actin Dynamics and Chondrogenic Function

**DOI:** 10.3390/ijms22063279

**Published:** 2021-03-23

**Authors:** Jasmin C. Lauer, Mischa Selig, Melanie L. Hart, Bodo Kurz, Bernd Rolauffs

**Affiliations:** 1 G.E.R.N. Research Center for Tissue Replacement, Regeneration & Neogenesis, Department of Orthopedics and Trauma Surgery, Faculty of Medicine, Medical Center—Albert-Ludwigs-University of Freiburg, 79085 Freiburg im Breisgau, Germany; jasmin.lauer@uniklinik-freiburg.de (J.C.L.); mischa.selig@uniklinik-freiburg.de (M.S.); melaniehar@gmail.com (M.L.H.); 2 Faculty of Biology, University of Freiburg, Schaenzlestrasse 1, D-79104 Freiburg, Germany; 3 Department of Anatomy, Christian-Albrechts-University, Otto-Hahn-Platz 8, 24118 Kiel, Germany; bkurz@anat.uni-kiel.de

**Keywords:** chondrocyte, cytoskeleton, stress fiber, SOX9, actin polymerization, actin depolymerization, pro-inflammatory cytokine signaling, growth factor signaling, fibrogenic expression profile, collagen aggrecan fragments

## Abstract

Numerous studies have assembled a complex picture, in which extracellular stimuli and intracellular signaling pathways modulate the chondrocyte phenotype. Because many diseases are mechanobiology-related, this review asked to what extent phenotype regulators control chondrocyte function through the cytoskeleton and cytoskeleton-regulating signaling processes. Such information would generate leverage for advanced articular cartilage repair. Serial passaging, pro-inflammatory cytokine signaling (TNF-α, IL-1α, IL-1β, IL-6, and IL-8), growth factors (TGF-α), and osteoarthritis not only induce dedifferentiation but also converge on RhoA/ROCK/Rac1/mDia1/mDia2/Cdc42 to promote actin polymerization/crosslinking for stress fiber (SF) formation. SF formation takes center stage in phenotype control, as both SF formation and SOX9 phosphorylation for *COL2* expression are ROCK activity-dependent. Explaining how it is molecularly possible that dedifferentiation induces low *COL2* expression but high SF formation, this review theorized that, in chondrocyte SOX9, phosphorylation by ROCK might effectively be sidelined in favor of other SF-promoting ROCK substrates, based on a differential ROCK affinity. In turn, actin depolymerization for redifferentiation would “free-up” ROCK to increase *COL2* expression. Moreover, the actin cytoskeleton regulates *COL1* expression, modulates COL2/aggrecan fragment generation, and mediates a fibrogenic/catabolic expression profile, highlighting that actin dynamics-regulating processes decisively control the chondrocyte phenotype. This suggests modulating the balance between actin polymerization/depolymerization for therapeutically controlling the chondrocyte phenotype.

## 1. Introduction

A range of cell types experience a modulated force balance between their endogenous cytoskeletal contractility and the external mechanical forces that are transmitted across cell-extracellular matrix (ECM) adhesions and into the cytoskeleton [1]. This intracellular force balance plays a key role in regulating basic cellular functions, such as cell proliferation, apoptosis, adhesion, and migration [2,3]. Importantly, it has been suggested that a pathological deregulation of this balance contributes to the pathogenesis of several human diseases [1]. Moreover, the etiology and/or clinical presentation of a wide range of diseases may result from abnormal mechanotransduction, and hence, anomalous processes by which cells convert perceived forces into a response [4].

Articular cartilage (AC) is an autonomously functioning connective tissue that absorbs and distributes the occurring mechanical joint loads. The resident cells of AC, the chondrocytes (CHs), are sparsely distributed within species-, joint type-, surface-specific [5,6,7] and diagnostically relevant [8,9] arrangements that are termed the superficial chondrocyte spatial organization (SCSO) and that can be used for spatial [10] and predictive modelling [11] in the context of a non-destructive quantitative optical biopsy for early disease detection. The CHs are quiescent, fully differentiated cells whose chondrogenic phenotype ensures a fine-tuned balance between anabolism and catabolism to maintain tissue homeostasis [12]. However, in osteoarthritis (OA), which is a common yet complex and not fully understood whole joint disorder, the phenotypic stability of the CHs is lost. This is believed to initiate and perpetuate a cascade of events that leads to permanent tissue damage [13] and disability [14,15,16]. Thus, many studies have focused on CH phenotype regulation in the context of hypertrophic differentiation [13,17], acute joint inflammation [18], aging and OA [19], mechanical/biophysical stimulation [20,21,22], inter-tissue communication [23], epigenetic regulation [24], in vitro chondrogenesis [25,26], therapeutic redifferentiation [27,28], and post-traumatic phenotype stabilization [29]. Collectively, these and many other studies have assembled a complex picture of how various extracellular stimuli and intracellular signaling pathways modulate the CH phenotype. However, despite these valuable insights, and because much of the knowledge pertaining to the field of mechanobiology has been gained from investigating other cell types than CHs, it remains unclear to what extent the processes that determine the CH phenotype are caused by or associated with the CH cytoskeleton and the signaling processes that originate from or converge on the cytoskeleton. As such information is not readily available but would be helpful to focus future research for advancing articular cartilage repair through controlling the CH phenotype, this review (i) summarizes the available knowledge on the crossroads between mechanobiology and the CH phenotype in detail; it (ii) elucidates how the CH phenotype is regulated by the cytoskeleton and the signaling processes that originate from or converge on the cytoskeleton; (iii) asks whether the major CH phenotype-regulating extracellular stimuli share a common cytoskeleton-associated pathway; and finally this review (iv) suggests a theory on how it is molecularly possible that the cytoskeletal changes that occur during CH dedifferentiation concomitantly cause decreased chondrogenic marker expression, whereas CH redifferentiation results in increased chondrogenic marker expression. This review presents the available data on specific topics in dedicated chapters that preserve as much original detail as possible to assemble a comprehensive picture of the signaling events associated with the cytoskeleton and the phenotype of CHs, whereas the discussion chapter focuses on interpreting these data.

## 2. Actin Isoforms, Classes, Nucleation, and Polymerization

Actin, the most abundant intracellular protein in eukaryotic cells [30], is involved in cell division, endocytosis, migration [31], and rapid signaling [32,33]. Six actin isoforms, coded by separate genes in mammals [34], are divided into three classes according to their isoelectric points, namely, α-, β-, and γ-actin. Generally, α-actin is found in muscle cells, whereas β- and γ-actin are found primarily in the cytoplasm of non-muscle cells [35]. The γ-actin can be considered a branched meshwork in a cortical and lamellar localization and β-actin an unbranched filamentous array, e.g., in stress fibers (SFs) [36]. Actin can exist as G-actin, which is a globular monomeric form, and as F-actin, the filamentous form of actin. For de novo filament formation, G-actin monomers first assemble into multimers (actin nucleation), which is regulated by the instability of actin dimers and the activity of actin-nucleating proteins, such as actin-related protein complex (Arp) 2/3, nucleation-promoting factors (NPFs), formins, and others [37]. F-actin consists of two polymer chains that are helically coiled around each other [38] and is polar with a plus (barbed) end, at which G-actin is rapidly added during polymerization, and a minus (pointed) end, at which G-actin is added slowly [39]. F-actin can degrade back into monomeric G-actin (actin depolymerization), which is regulated by actin-depolymerizing factor (ADF), cofilin, and others [40]. Table 1 lists the definitions of the terms. SFs consist of 10–30 F-actin bundles [41] crosslinked by various proteins such as α-actinin [42] and filamin [43], among others, and/or non-muscle myosin II (NMMII) [44]. SFs can contain both F-actin and myosin and then are referred to as actomyosin bundles [32], whereas other, non-contractile SFs do not contain myosin [45,46,47,48]. When assembling this review, the authors noted that SFs were visualized in many studies by using only phalloidin, which depicts F-actin. Thus, in many studies the term “SF” does not differentiate between SFs containing F-actin vs. F-actin and myosin. The present study follows this approach. Interestingly, the SF orientation determines the contractile properties of a cell [49]. This is supported by a finite element modeling study on live vascular smooth muscle cells (SMCs), which demonstrated that stress distributions within the actin SF network are based more on the network’s geometry than on the number or thickness of the SFs [50].

## 3. Actin Stress Fiber Classification Systems

Two SF classification systems are established. In polar motile cells, such as fibroblasts, SFs are classified by their subcellular localization into (i) ventral SFs; (ii) dorsal SFs; (iii) transverse actin arcs; and (iv) perinuclear actin caps [32,48,49,51,52,53,54]. Ventral SFs, the most abundant and contractile SF type, span across the cell base from one side to the other and are attached at each end to focal adhesions (FAs) [49,55,56] and are proposed to be the main SFs involved in mechanosensing and mechanotransduction [49]. Dorsal SFs are non-contractile [56] due to the lack of myosin [45,46,47,48] and are bound with one end to FAs at the ventral side, whereas the other end points towards the dorsal side of the cell, together forming a loose network at the center [49,56,57]. On a side note, the definition of a ventral vs. dorsal cell side is derived from the dorsal/ventral embryonic patterning, whereas “apical/basal” describes, e.g., epithelial cell polarity with regard to their barrier function [58]. Generally, it appears that ventral and basal or dorsal and apical are used synonymously. Moreover, cytoskeletal studies used the term “ventral” for describing the material-facing, adhering cell side [47]. Transverse actin arcs are curve-shaped actomyosin bundles [45] that contain myosin IIB and α-actinin [52] but are not bound to FAs [56,59]. Actin arcs are connected to dorsal SFs [32] and travel along the dorsal SFs towards the cell center [49,56,57]. Interestingly, dorsal SFs and transverse arcs assemble to an interconnected contractile matrix promoting the formation of perinuclear actin cap fibers that induce nuclear reorientation [48,60]. Noteworthy, immotile cells often display only ventral SFs [54].

Another classification system that applies to most cell types separates the SFs into (i) peripheral and (ii) central SFs [54,61,62,63,64,65]. These two SF types can sustain different mechanical stress levels [54,65,66,67] and have different viscoelastic properties and myosin activators [54,61,68,69]. Peripheral SFs are strained to a vast extent, due to a higher plateau retraction distance and lower elasticity, compared to central SFs [54,64]. When attempting to match the SF classification systems, peripheral SFs can be assigned to ventral SFs, whereas central SFs can be designated to ventral, dorsal SFs, and transverse arcs [54]. To our knowledge, no study has yet investigated CH SFs in this context and only a few studies even quantified the CH F-actin content, e.g., in knee AC pig CHs (pCHs) [70], AC bovine CHs (bCHs) from metacarpophalangeal joints (MCPJ) [71], and sternal chicken CHs (cCHs) [72].

## 4. Regulation of Actin Dynamics

**Figure 1:**
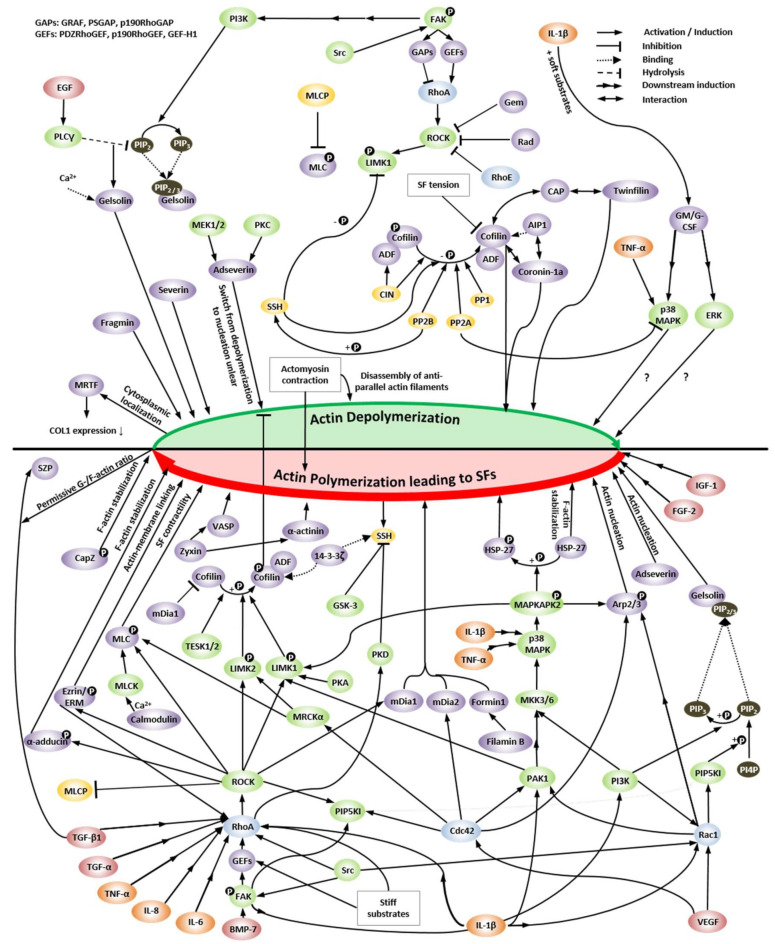
Regulation of actin dynamics. The relevant signaling pathways and molecules for regulating actin depolymerization (top, green half circle) compared to actin polymerization and/or stress fiber (SF) formation (bottom, red half circle), including upstream signaling through pro-inflammatory cytokines and growth factors as well as downstream signaling to actin-modulating proteins, e.g., cofilin and gelsolin, are illustrated. The legend in the upper right corner details the symbols used; the dotted arrow termed “binding” describes the binding of a substance, which might cause either inhibition or activation. Details are specified in the text. Abbreviations: ADF: actin-depolymerizing factor, AIP1: actin-interacting protein 1, Arp2/3: actin-related protein 2/3, BMP-7: bone morphogenetic protein 7, CAP: cyclase-associated protein, CIN: chronophin, COL1: type I collagen, DEX: dexamethasone, EGF: epidermal growth factor, ERK: extracellular signal-regulated kinase, ERM: ezrin/radixin/moesin, FAK: focal adhesion kinase, FGF-1: fibroblast growth factor 1, FGF-2: fibroblast growth factor 2, GAPs: GTPase-activating proteins, GEFs: guanine-nucleotide exchange factors, G(M)-CSF: granulocyte(-macrophage) colony-stimulating factor, GSK-3: glycogen synthase kinase 3, HSP-27: heatshock protein 27, IL-1β: interleukin 1β, IL-6: interleukin 6, IL-8: interleukin 8, LIMK1/2: LIM kinase 1/2, MAPK: mitogen-activated protein kinase, MAPKAPK2: MAPK-activated protein kinase 2, MEK1/2 / MKK3/6: MAPK kinase 1/2 / 3/6, MLC: myosin light chain, MLCK: MLC kinase, MLCP: MLC phosphatase, MRCKα: myotonic dystrophy-related Cdc42-binding kinase α, MRTF: myocardin-related transcription factor, PAK1: p21-activated kinase, PI4P: phosphatidylinositol-4-phosphate, PIP2: phosphatidylinositol (4,5)-bisphosphate, PIP3: phosphatidylinositol (3,4,5)-trisphosphate, PI3K: phosphatidylinositol 3-kinase, PIP5KI: phosphatidylinositol 5-kinase type I, PKA/PKC/PKD: protein kinase A/C/D, PLCγ: phospholipase Cγ, PP1/PP2A/PP2B: protein phosphatase type 1/ 2A/ 2B, PTHrP: parathyroid hormone-related protein, ROCK: Rho-kinase, SOX9: SRY-box transcription factor 9, SSH: slingshot phosphatase, SZP: superficial zone protein, TESK1/2: testicular protein kinase 1/2, TGF-α/β1: transforming growth factor α/β1, TNF-α: tumor necrosis factor α, VASP: vasodilator-stimulated phosphoprotein, VEGF: vascular endothelial growth factor, YAP: Yes-associated protein.

Later text sections demonstrate that cytoskeletal composition and mechanics and specifically SF formation and disassembly affect CH function and perhaps even joint health. Thus, the molecular mechanisms that govern SF assembly take center stage in the effort to better understand and control the CH phenotype and are reviewed here. In this context, most studies have mainly focused on cells other than CHs. Actin dynamics are centrally regulated by the small GTPases RhoA, Rac1, and Cdc42 [73] (Figure 1). RhoA is one of the main regulators of SF assembly [74] and promotes SF formation through its effectors, ROCK and the formin mammalian Diaphanous 1 (mDia1/DIAPH1/DRF1) [75,76]. Moreover, RhoA signaling additionally regulates the transcription of several genes that encode cytoskeletal proteins and, thus, controls actin cytoskeleton composition [77,78]. The RhoA effector mDia1 produces actin filaments by actin nucleation and polymerization and facilitates long parallel actin filaments relevant for the formation of dorsal SFs [46,79]. Interestingly, the actin nucleation activity of mDia1 is enhanced by transiently increased cytoplasmic G-actin through SF disruption and actin turnover, independently of Rho or Ca^2+^ [80]. Moreover, mDia1 assembles F-actin by helical rotation, which makes F-actin more resistant to binding and severing of cofilin through untwisting the long-pitch helix of F-actin [81]. However, mDia formins are not the only members of the formin homology family proteins that are able to regulate the actin turnover, as a filamin B physically interacts with formin1 to regulate formin1 function [82]. Interestingly, G-actin also regulates megakaryoblastic leukemia 1 (MAL) [83], a coactivator of the transcription factor serum response factor (SRF), which is essential for the remodeling of the actin cytoskeleton during cell motility [84]. SRF is also relevant in the context of enhancing CH type I collagen (*COL1*) expression [85].

The RhoA effector ROCK inhibits ADF- and cofilin-mediated disassembly of actin filaments through phosphorylation/activation of LIM domain kinase 1 and 2 (LIMK1, LIMK2), which increases the phosphorylation and, thus, inhibition of cofilin [86] for stabilizing existing actin filaments [86,87]. Cofilin inhibition has also been linked via LIMK2 to Rho and Cdc42 [88], via LIMK1 to Rac [89,90], and to 14-3-3ζ, a conserved regulatory molecule, through preventing the dephosphorylation/activation by binding phospho-cofilin [91] and slingshot (SSH) family phosphatases [92]. In turn, the cofilin regulating LIMKs are phosphorylated by myotonic dystrophy-related Cdc42-binding kinase alpha (MRCKα), a downstream effector of Cdc42 [93], one of the small GTPases. LIMK1 also appears to be regulated by the p21-activated kinase 1 (PAK1)-mediated pathway [94]. In addition to regulating cofilin, ROCK also phosphorylates/activates myosin light chain (MLC) for cross-linking and induction of actomyosin bundles, SF formation, and contractility [87]. The assembly and polymerization of cortical SFs is furthermore supported by the ROCK-mediated phosphorylation/activation of the ERM family proteins (ezrin/radixin/moesin) that link membrane-associated proteins to actin filaments at the cell cortex [95,96,97,98]. Thus, several proteins involved in regulating actin-filament assembly and contractility are phosphorylated by ROCK, making ROCK the major regulator of actin-cytoskeleton assembly and cell contractility [99] (Figure 1). In most cases, the consensus amino-acid sequences for phosphorylation of substrates by ROCK are R/KXS/T or R/KXXS/T [94,100].

In contrast to RhoA and its effectors, the other small GTPases Rac1 and Cdc42 coordinate SF assembly more indirectly. Rac1 activates the actin nucleating complex Arp2/3, whereas Cdc42 promotes actin polymerization through mDia2 [101]. Both Arp2/3- and mDia2-nucleated filaments can be considered as “building blocks” for contractile SFs [32]. Located downstream of Rac1 and Cdc42, activated/phosphorylated F-actin side-binding heat shock protein 27 (HSP-27) also supports actin polymerization by dissociating from F-actin [102,103,104,105]. HSP-27 is phosphorylated by MAPKAPK2, which (i) is part of the p38 MAPK pathway downstream of Rac1 and Cdc42 [106]; and (ii) phosphorylates/activates LIMK1 [107] and p16-Arc, a part of the Arp2/3 complex [108,109]. Moreover, Rac1 induces the activation of mitogen-activated protein kinase kinase (MKK) 3 and MKK6 for activation of p38 MAPK [110,111,112] to phosphorylate HSP-27 for supporting actin polymerization. Interestingly, vascular endothelial growth factor (VEGF) also induces actin polymerization through the p38-MAPKAPK2–LIMK–cofilin [107] and p38-MAPKAPK2–HSP-27 pathways [104] and signals upstream of p38 through MKK3 [110] and PAK1 [106], which is activated by Cdc42 [106,113] and Rac [106,114].

Actin depolymerization through cofilin dephosphorylation/activation is induced by SSH phosphatases [115] that also dephosphorylate LIMK1 [116], protein phosphatases type 1 (PP1) [117,118], type 2A (PP2A) [117,119], type 2B (PP2B, calcineurin) [118], chronophin (CIN) [120], and actin-interacting protein 1 (AIP1) [121,122,123,124]. The three component system, composed of cofilin, AIP1, and coronin, rapidly disassembles the actin filaments from both ends in bursts [125]. Cyclase-associated protein (CAP) interacts with cofilin to speed up the depolymerization of the actin filament pointed ends from 100- to 330-fold [126,127]. CAP, on the other hand, also works together with twinfilin for acceleration of actin filament disassembly by 3-fold on the barbed end or 17-fold on the pointed end [128]. Twinfilin supports the dissociation of the capping protein from the barbed ends, inducing actin depolymerization by 6 subunits per second [129]. Actin depolymerization is also mediated via gelsolin activity, which is regulated by Ca^2+^ levels [130,131] and phosphatidylinositol(4,5) biphosphate (PIP_2_) [131,132] and phosphatidylinositol (3,4,5)-trisphosphate (PIP_3_) [133]. A high Ca^2+^ level induces gelsolin activity, causing actin depolymerization, severing, and barbed-end capping [130]. Through binding of PIP_2_ or PIP_3_, gelsolin is inactivated, which not only stops severing but also re-induces actin polymerization through actin uncapping [131,133,134,135]. PIP_2_ is (i) produced by the phosphatidylinositol 4-phosphate 5-kinase type I (PIP5KI) isoforms α, β, and γ, which are regulated by the small GTPases Rho via ROCK, Rac, and Cdc42 [136,137]; is (ii) regulated through phosphatidylinositol-3-kinase (PI3K), which phosphorylates PIP_2_ to produce PIP_3_ [138]; and (iii) through phospholipase Cγ (PLCγ), which is a downstream effector of epidermal growth factor (EGF) and hydrolyzes PIP_2_ [124,131,138,139]. A cofilin-centered switch from actin depolymerization to polymerization is the phosphorylation/inhibition of SSH family phosphatase activity by GSK-3 [140] and protein kinase D type 1 (PKD1) and type 2 (PKD2) [141,142]. Actin depolymerization may also be regulated positively as well as negatively by mechanical forces. The binding rate of cofilin to F-actin is decreased when the SFs are tensed at a low level (>2 pN) between tweezers compared to SFs in solution, leading to a decrease in actin severing [143]. In contrast, another study has shown that filament tension up to 13 pN has no significant effect on the cofilin severing activity. However, cofilin binding causes a mechanical torque of crosslinked interconnected actin filaments, which enhances actin severing drastically up to 100-fold in case the filaments are saturated with cofilin [144]. Moreover, myosin disassembles antiparallel actin filaments, but not parallel F-actin, indicating that actomyosin contraction can also cause depolymerization for spatial remodeling of the actin cytoskeleton [145]. In contrast, another study has shown that actomyosin contraction induces F-actin stabilization in lamellipodia and lamella [146].

The RhoA/ROCK pathway mediates directly or indirectly ventral, dorsal, and transverse SF formation. Ventral SF and transverse arc actomyosin contractility results either from ROCK-mediated MLC phosphorylation and/or inhibition of the phosphorylation of the myosin light chain phosphatase (MLCP) [74,147,148,149,150,151], or through Ca^2+^/calmodulin-mediated activation of myosin light chain kinase (MLCK) and subsequent MLC phosphorylation [32]. Dorsal SF assembly is induced by RhoA-mediated mDia activation for nucleation and polymerization [32,46,74,152] (Figure 1). Moreover, the RhoA/ROCK pathway also impairs vimentin filament formation via ROCK2-mediated phosphorylation of several sites in vimentin [32] and likely affects vimentin network-mediated cell stiffness. Proteins like NMMIIB and MLCK are predominantly localized to peripheral SFs, while NMMIIA and ROCK are primarily localized to central SFs [54,61,62].

The RhoA/ROCK pathway is activated through focal adhesion kinase (FAK), whose autophosphorylation at FAK^Y397^ exposes a binding site for Src [153]. This results in a Src-dependent phosphorylation of FAK at FAK^Y576^ and FAK^Y577^ and subsequent FAK activation [154]. The downstream effects of activated FAK on Rho (and Cdc42) signaling are mediated by so-called GTPase-activating proteins (GAPs), such as (i) GTPase regulator associated with FAK (GRAF); (ii) PH- and SH3-domain-containing RhoGAP protein (PSGAP); and (iii) p190RhoGAP, which hydrolyze GTP to GDP for inhibition of Rho. In contrast, located downstream of activated FAK, Rho-activating guanine-nucleotide exchange factors (GEFs), such as (i) PDZRhoGEF and (ii) p190RhoGEF, which exchange GDP with GTP, activate Rho signaling. These mechanisms are reviewed in more detail in [155] and suggest that FAK might alternately control the activation and inactivation of Rho [156]. Moreover, it appears that Rho is associated with SF formation and Cdc42 with filopodia formation [155]. Other small GTP-binding proteins, such as Gem and Rad, are negative regulators of ROCK [157] or, in the case of RhoE, inhibit ROCK1 [158]. Interestingly, on harder substrates, increased RhoA expression and activation and increased ROCK activity [159] and increased activity of GEF-H1 [160] were observed, compared to less stiff substrates, indicating (co-) regulation of the RhoA/ROCK pathway through mechanical cues. Activated GEF-H1 also connects microtubule dynamics to Rho-dependent SF formation and contractility [161], illustrating how mechanical cues affect cytoskeletal contractility. In addition to controlling Rho signaling, FAK functions in the regulation of Cdc42 and Rac activity for control cellular polarization, the extension of lamellipodia, and cell migration through the modulation of a so-called paxillin kinase linker (PKL/Git2)–β-pix complex [155]. On a side note, the GEF that activates Cdc42, termed FGD1, not only regulates the actin cytoskeleton [162], but is also the locus for facio-genital dysplasia (Aarskog–Scott syndrome) [163,164], highlighting the in vivo relevance of the regulation of actin dynamics.

## 5. Cytoskeletal Proteins and Associated Signaling Pathways in Passaging-Induced Chondrocyte Dedifferentiation

Serial passaging, also termed expansion, is a well-accepted model of CH dedifferentiation, in which passaging induces a loss of phenotype, morphology, and elastic and viscoelastic properties. Many effects are cartilage tissue zone-specific, occur rapidly within one passage, and produce a more homogeneous and dedifferentiated phenotype that is drastically different from the initial differentiated state [165,166,167,168]. Differentiated CHs have a round and spheroid shape, small diameter [169], small spreading area, and a low elongation factor [170], whereas dedifferentiated CHs have in vitro an amoeboid and fibroblast-like shape [71,169,171], large diameter [169], large spreading area, a high elongation factor [170], and a reduced amount of primary cilia [172]. With increasing passage, the CHs become more motile and travel a longer distance [170]. Generally, CHs in higher passages, e.g., ≥P5, are considered to have lost their phenotype, as CHs from passages P1 to P4 can be induced to regain a CH phenotype via high density culture or using polymer scaffolds [168]. P5 to P8 CHs have not been shown to produce a CH-specific extracellular matrix upon attempted redifferentiation through a high-density culture, an alginate bead culture, or a culture on biodegradable polymer scaffolds [167,168,173]. Specifically *type II collagen* (*COL2*) expression, which has been linked to the cytoskeleton [174], steadily declines during passaging with production, essentially ceasing in P5 [165].

It has been known for a long time that passaging of CHs induces SFs, as demonstrated in cCHs [175]. One study described already in 1988 that SF modification, termed F-actin microfilament modification, is a sufficient signal for *COL2* re-expression and also mediates the effects of cell shape rounding [174]. More recently, CH dedifferentiation has been associated specifically with increased actin polymerization, leading to SFs. Passaging-induced dedifferentiation of MCPJ AC bCHs in 2D culture caused an increased gene expression of the proliferation-associated molecules cyclin D1 and ki67 and *COL1* (10640-fold increase), a more than approximately 5-fold decreased *COL2* and aggrecan gene expression, markers of a healthy phenotype, and also an enlarged, more elongated morphology with increased actin polymerization, when comparing P0 to P2 [71]. Interestingly, the knockdown of the actin depolymerization factor cofilin induced further actin polymerization in P2 AC bCHs from MCPJ and increased *COL1* gene expression. The pharmacological depolymerization of actin using cytochalasin D, an inhibitor of actin polymerization that binds to the barbed ends of F-actin, re-induced aggrecan (but not *COL2*) expression and decreased *COL1* expression. In the same study, subsequent 3D culture of 2D-passaged P2 AC bCHs from MCPJ resulted in actin depolymerization and decreased *COL1* gene expression but had no effect on aggrecan or *COL2* expression. Further addition of cytochalasin D enhanced these results but had no effects on *COL2*. Other agents, such as phalloidin, nocodazole, or paclitaxel, had no additional effects on aggrecan, *COL1*, or *COL2* gene expression, pinpointing that actin polymerization via 3D culture modulates *COL1* expression during dedifferentiation. Comparable phenotype-reversing effects of cytochalasin D were also observed in monolayer-passaged AC bCHs from knee joints [176].

Comparing the actin cytoskeleton in both primary and dedifferentiated P5 sternal cCHs cultured on plastic, P0 sternal cCHs displayed low levels of diffusely staining cytoplasmic actin, whereas serially passaged and simultaneously fibroblast growth factor 2 (FGF-2)-treated dedifferentiated P5 sternal cCHs showed prominent SFs [72]. This dedifferentiation of initially round primary sternal cCHs into a fibroblast morphology in P5 correlated with RhoA induction, actin polymerization, and SF formation. A subsequent alginate culture of the P5 sternal cCHs for inducing redifferentiation led to a loss of both RhoA and SFs, together with the re-expression of CH-specific markers. These studies clearly established in the context of CH passaging that actin polymerization is associated with CH dedifferentiation and actin depolymerization with CH redifferentiation.

Another study that focused on P0 vs. P2 AC bCHs from MCPJ and the actin-binding protein adseverin (scinderin) reported a loss of the adseverin protein already at day 14 and subsequently in P2 [177]. Adseverin knockdown by small interfering ribonucleic acid (siRNA) increased the cell size and elongation, the number of actin-free barbed ends, and SF formation in P0 AC bCHs from MCPJ. Additionally, the expression of vinculin and α-smooth muscle actin and the ability to contract collagen hydrogels was increased, whereas adseverin overexpression via transfection in P2 AC bCHs from MCPJ partially reversed these changes, upregulated aggrecan and SRY-box transcription factor 9 (*SOX9*) expression, and downregulated *COL1* expression but also maintained low *COL2* levels. On a side note, SOX9 is a transcription factor essential for the formation of all cartilaginous tissue that targets the gene *COL2A1* for COL2 [178]. Because adseverin can promote F-actin depolymerization, e.g., as demonstrated in [179], but also actin nucleation depending on the intracellular conditions [180], this study illustrates that modification of the CH actin cytoskeleton via alterations in actin-binding proteins affected not only the cytoskeleton itself but also the cell shape, size, mRNA expression profile, and contractility.

In [181], one-month-old AC knee joint *rabbit* CHs (rabCHs) were passaged to P2 and then underwent siRNA knockdown of zyxin, a mechanosensitive protein that rapidly relocates from FAs to actin SFs in response to mechanical cues [182,183] at sites of strain-induced stress fiber damage, and that is essential for SF repair and generation of traction force [183]. Because zyxin recruits actin polymerization factors, such as vasodilator-stimulated phosphoprotein (VASP), and actin cross-linkers, such as α-actinin [183,184], zyxin knockdown effectively weakens actin polymerization. In comparison to untreated controls, zyxin knockdown led in [181] to reduced SFs and an increased G/F-actin ratio in P2 AC rabCHs, confirming an actin depolymerization-decreasing effect of zyxin knockdown. Interestingly, this led additionally to a markedly reduced expression of COL2 and type X collagen (COL10) but not COL1 on both the protein and mRNA levels. Interestingly, the authors of [181] noted that the expression of COL2 was mainly affected when the zyxin level was lower than the baseline levels and the expression of COL1 was largely increased when the F-actin level was higher than the baseline levels. In part, this can be explained by the fact that zyxin is a regulator of SF mechanics. In zyxin absence, the SFs become fluid-like [185], suggesting that the SF force-sensing mechanisms, e.g., in FAs, would receive decreased input. This would affect, e.g., the activation of the RhoA/ROCK pathway through FAK and Src [186,187,188] and, in turn, decrease SOX9 phosphorylation by ROCK.

One study presented passage-specific changes in the CH phenotypic characteristics, such as changes in *COL1*, *COL2*, aggrecan, *SOX9* mRNA expression, cell morphology, and motile traits, as well as alterations in FA area and length, and the total FAK, vinculin, α-actinin, paxillin, RhoA, Rac1, and Cdc42 protein expression characteristics in serially passaged 4–6-week-old hip joint AC *rat* CHs (rCHs) [170]. After passaging, P4 AC rCHs were treated with the FAK inhibitor PF573228, the ROCK inhibitor Y27632, the Rac1 inhibitor NSC23766, or the Src inhibitor 4-amino-5-(4-chlorophenyl)-7-(dimethylethyl)pyrazolo[3,4-*d*]pyrimidine (PP2). Compared to dedifferentiated but untreated P4 AC rCHs, the spreading area was rescued with the FAK inhibitor, whereas cell elongation was decreased with the FAK, ROCK, and Rac1 inhibitors, with the ROCK inhibitor being most effective. In terms of motility, the FAK, ROCK, and Rac1 inhibitors all also somewhat decreased the travelled distance of the CHs. Interestingly, *COL2* expression was rescued with all four inhibitors and to comparable extents, illustrating the profound effects of FAK, Src, ROCK, Rac1, on *COL2* expression. *SOX9* mRNA expression, which was reduced by approximately 10-fold in P4 AC rCHs compared to P0 AC rCHs, was recovered to more than 50% by using the FAK inhibitor PF573228 or the Src inhibitor PP2, which had the stronger effect. In contrast, the ROCK and Rac1 inhibitors had no effects on *SOX9* expression. This is in contrast to other studies, which demonstrated that ROCK inhibition rescued *SOX9* expression [189,190]. We explain this discrepancy by differences in inhibitor concentrations and incubation times: [170] used 10 µM Y27632 for ROCK inhibition for 48 h, whereas [189] used 100 µM Y27632 for 72 h and [190] used 10 μM Y27632 for 72 and 96 h, indicating that longer incubation times are necessary for rescuing *SOX9* expression by ROCK inhibition. With regard to [170], it would have been interesting to see the effects on SOX9 phosphorylation but this topic was not examined. However, aggrecan mRNA expression was similarly rescued by Src or FAK inhibition but to a smaller extent than *SOX9*, and ROCK and Rac1 inhibitors had no rescuing effect on aggrecan. However, the ROCK inhibitor and to a smaller extent the Rac1 inhibitor led to increased *COL1* expression. Whether the ROCK inhibitor had more effects in increasing *COL1* or *COL2* cannot be answered, as the results were presented relative to the controls. Overall, the *SOX9* rescue constituted the most successful recovery. However, the resulting expression levels never fully reached those of the P0 CHs, regardless of using Src or FAK inhibitors [170]. This is somewhat expectable, as, e.g., *COL2* expression essentially ceases in P5 [165] and P5 to P8 CHs cease to produce CH-specific markers upon attempted redifferentiation [167,168]. Lastly, a study [170] confirmed these results using FAK siRNA transfection, which largely confirmed the pharmacologically achieved results and, additionally, revealed that using FAK siRNA had increasing effects on *COL1* expression, whereas FAK inhibition did not alter *COL1* expression.

**Figure 2:**
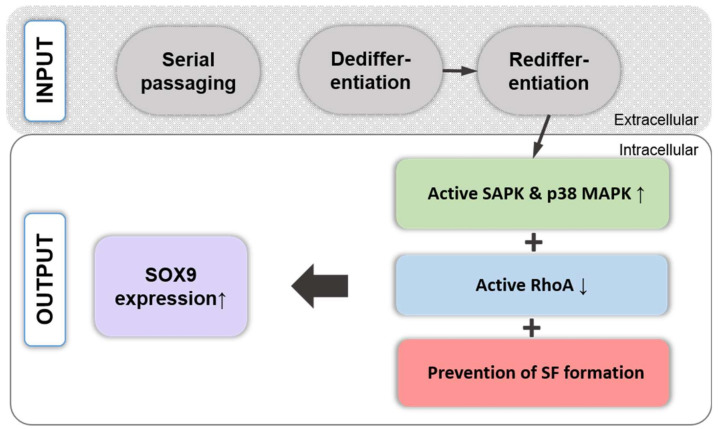
Correlation between actin polymerization, dedifferentiation, and redifferentiation. Redifferentiation of dedifferentiated CHs induced by serial passaging depends on active SAPK and p38 MAPK, low active RhoA, and prevention of SFs, resulting in increased *SOX9* expression. The up and down arrows indicate an increase or decrease.

In 2006, a study with a mechanistic focus demonstrated that endogenous *SOX9* mRNA expression was 10-fold lower in P2 than P0 AC human CHs (hCHs) from knee joints, and that *SOX9* expression can be rescued using a redifferentiation-inducing 3D alginate culture, monolayer CHs treated with cytochalasin D, or ROCK inhibition [189]. However, the re-induced increase in SOX9 mRNA expression was accompanied by both cell rounding and SF disruption. Subsequently, the study then dissected the effects of rounding vs. loss of SFs by inhibiting actin polymerization with cytochalasin D and SF formation via ROCK inhibition with Y27632 after 72 h of incubation. Interestingly, both substances increased *SOX9* mRNA expression at their two highest concentrations, at which also SF loss was observed. However, using 10 µM Y27632 led additionally to an elongated CH shape, whereas 100 µM Y27632 led to a round shape, suggesting that the rounding up was less important for *SOX9* re-expression than SF loss because *SOX9* re-expression occurred already in elongated CHs at 10 µM Y27632. The authors [189] then tested the effects of the protein synthesis inhibitor cycloheximide (CHX), which activates the stress-activated protein kinase (SAPK) pathways p38 mitogen-activated protein kinase (MAPK) and JUN N-terminal kinase (JNK) [191,192,193,194]. On a side note, this activation through an inhibitor occurs in a process termed superinduction, in which protein synthesis inhibitors paradoxically increase the expression of early-gene products, such as the SAPK pathways or various cytokines [191]. For example, in CHs, IL-1β and TNF-α signal through the p38 MAPK pathway [195]. In [189], CHX superinduced *SOX9* expression in alginate-cultured CHs almost 5-fold after 24 h and 20-fold after 48 h. However, this superinduction was not successful in monolayer-cultured CHs, except when cytochalasin D or Y27632 were used in addition to using CHX. This combination increased *SOX9* by 4-fold, suggesting that prevention of SF formation was necessary for induction. For mechanistic experiments, subsequent usage of SB202190 for inhibiting the p38 MAPK pathway and separate usage of SP600125 for inhibiting the JNK pathway in monolayer-cultured cells, again in the presence of CHX for superinduction, and also in the presence of Y27632 to inhibit SF formation, demonstrated that CHX superinduction of *SOX9* mRNA expression was detectable after only 5 h. Moreover, these inhibitor experiments revealed that *SOX9* expression was p38 MAPK- but not JNK-dependent. A similar setup using hCHs cultured for 2 days in 3D alginate confirmed the p38 MAPK dependence of *SOX9* expression superinduction. Interestingly, compared to day 0, after two days of alginate culture, *SOX9* upregulation was also induced, but to a smaller extent than under CHX treatment. Notably, the usage of SB202190 for inhibiting the p38 MAPK under CHX and Y27632 treatment did not affect the alginate culture-induced upregulation of *SOX9* expression, suggesting a different mechanism that was likely associated with the cell rounding usually observed in alginate [189]. In this context, another study that is discussed further below in detail [72] observed that a 3D alginate culture of dedifferentiated P5 cCHs derived from embryonal sterna led to loss of SFs and also to loss of total and active RhoA protein. Moreover, they showed that monolayer-expanded, dedifferentiated P4 sternal cCHs that were subsequently cultured in 3D alginate exhibited increased the SOX9 protein expression, compared to P4 sternal cCHs cultured on plastic. Additionally, in monolayer P5 sternal cCHs cultured on plastic, the pan-Rho antagonist Tat-C3 transferase led to increased *SOX9* expression as well as increased *COL2* and aggrecan expression, illustrating a causal connection between *SOX9* expression and low total and active RhoA protein levels induced in CHs by a 3D alginate culture or by C3 transferase. This is supported by a study [190] that demonstrated that, in ATDC5 cells, ROCK inhibition with 10 μM Y27632 resulted in a 2-fold increase in the *SOX9* mRNA levels at days 3 and 4, RhoA overexpression reduced the *SOX9* mRNA levels by day 6, and ROCK inhibition rescued this effect exhibited by a 2-fold increase in *SOX9* mRNA expression. Thus, the fact that in [189] a basal *SOX9* expression was not affected by the usage of SB202190 for inhibiting p38 MAPK signaling (under CHX and Y27632 treatment) can be explained by a 3D alginate-associated increase in *SOX9* expression, mediated via low RhoA/ROCK levels in 3D alginate, which is consistent with [72]. Thus, in the context of CH passaging, dedifferentiation, and redifferentiation, the molecular context of how actin polymerization is connected to CH dedifferentiation and actin depolymerization with CH redifferentiation is that the *SOX9* expression is induced by SAPK/p38 MAPK activity [189], but requires prevention of SF formation and low active RhoA protein levels [72,190] (Figure 2).

## 6. Stress Fibers in Chondrocyte Differentiation and Dedifferentiation

**Figure 3:**
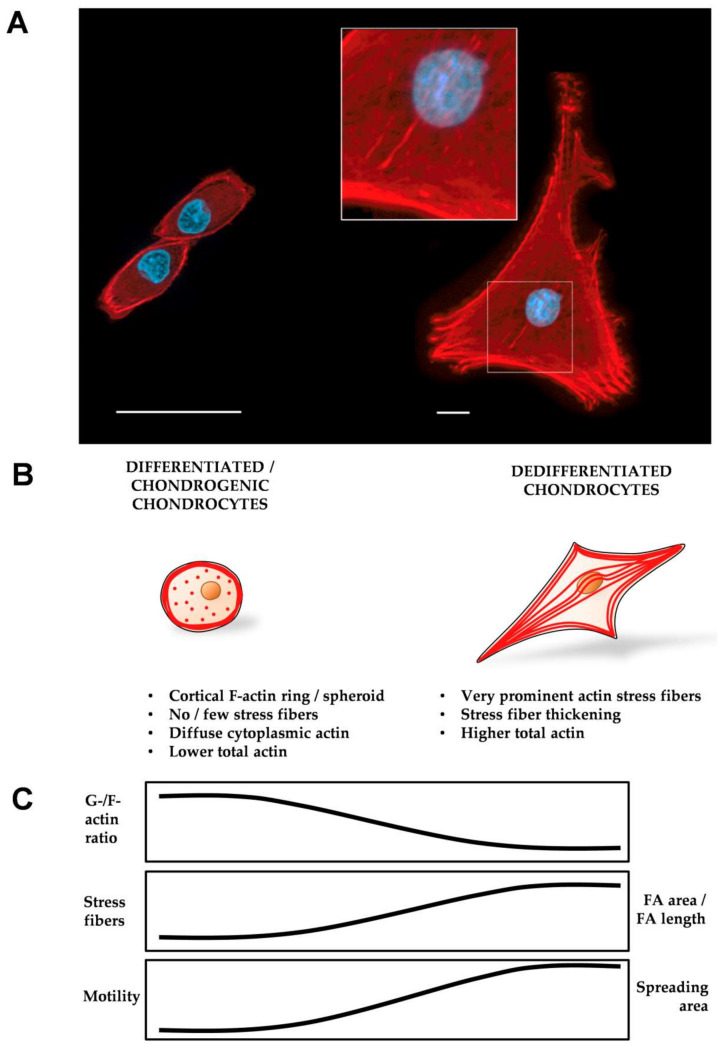
(**A**) Representative 40x images of primary hCHs with different morphologies and the F-actin distribution on day 1 of cultivation, acquired with an AxioObserver-Z1, Zeiss, Germany. Left side: CHs cultured on a glass substrate coated with fibronectin. The image was taken with oil immersion. Right side: a CH cultured on a tissue culture polystyrene substrate. Red: F-actin, blue: nucleus. Scale bar: 50 µm. (**B**) Schematic actin organization, FA area, spreading area, and motility related to the CH differentiation status. Differentiated and round CHs have a cortical F-actin spheroid that appears as a “ring” in 2D and punctate (freckled) actin in the cytoplasm [70,71,72,169,170,175,196], and have low total actin. (**C**) A high G-/F-actin ratio [71], little or no SFs [70,71,72,196], a small FA area, small spreading area, and low motility [170] characterize differentiated CHs. Dedifferentiated, fibroblastic CHs have prominent, thick SFs [70,71,72,170,175,196], a high total actin, a low G-/F-actin ratio [71], a large focal adhesion (FA) area, large spreading area, and high motility [170].

Whereas the previous text section summarizes the cytoskeletal protein alterations and the associated signaling events that occur in passaging-induced CH dedifferentiation, this section condenses the accompanying SF-specific alterations. Differentiated AC pCHs, AC bCHs, sternal cCHs, and AC rCHs display a cortical F-actin ring with intense staining at the cell periphery and a diffuse, punctate, and evenly distributed cytoplasmic actin staining [70,71,72,169,170,175,196], e.g., when CHs are either investigated directly after isolation or after a short time, 3D, or a micromass culture (Figure 3A,B). Interestingly, the cortical F-actin “ring” appears as a ring in 2D but is more accurately described as spheroidal in nature [197].

**Figure 4:**
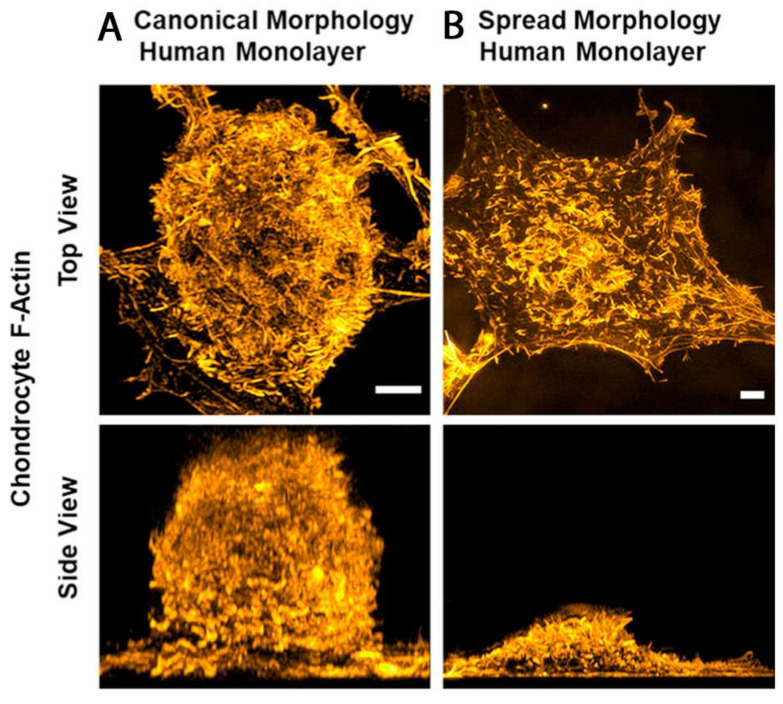
(**A**) Rounded vs. (**B**) spread morphologies of primary hCHs imaged in super-resolution using 3D structured illumination microscopy (3D-SIM) on a GE DeltaScan OMX SR microscope. Isolated primary AC hCHs from ankle joints were plated on a fibronectin (FN)-coated cover glass using standard 2D cell culture techniques, fixed with 4% paraformaldehyde, stained with ActinRed 555 Ready probes Reagent (ThermoFisher), and imaged. The maximum intensity projections of the volumetric image stacks are shown. Scale bars: 2 μm. The images are reprinted (adapted) with permission from [197]. Copyright (2020) American Chemical Society. We also received reprint permission from Scott T. Wood [197].

Importantly, differentiated CHs do not appear to exhibit SFs at all. Instead, they display a high G-/F-actin ratio and low total actin amount, which was demonstrated in AC pCHs, AC bCHs, sternal cCHs, and AC bCHs [70,71,72,196] (Figure 3B,C). In strong contrast, dedifferentiated AC pCHs, AC bCHs, sternal cCHs, and AC rCHs, generated by a prolonged culture time, serial monolayer expansion, or the addition of the pro-inflammatory cytokine interleukin-1 (IL-1), have prominent, thick SFs [70,71,72,170,175,196] that are located beneath the cell membrane closest to the substrate, have punctate (freckled) actin distant from the substrate [169], and have a low G-/F-actin ratio as well as high total actin amount [71,169]. Ventral SFs can be observed in all dedifferentiated CHs except when dedifferentiation (of AC pCHs or AC hCHs) is induced by the pro-inflammatory cytokine IL-1 and leptin [70,171], a hormone associated with obesity in women and a catabolic role in AC [198]. In some studies using AC rCHs, SFs below the nucleus were seen in random orientation [170], which were classified as dorsal SFs. These findings are summarized in Figure 3. Figure 4 shows two hCH morphology examples of primary AC hCHs isolated from an ankle joint that were imaged in super-resolution using 3D structured illumination microscopy (3D-SIM), illustrating the F-actin differences as a function of rounded vs. spread morphologies.

We propose using the “ventral/dorsal/transverse” SF classification system in the context of dedifferentiated, fibroblast-like CHs, whereas the “peripheral/central” SF classification system may be more suitable for the differentiated, round CH phenotype.

## 7. Cytoskeletal Differences between Healthy and OA Chondrocytes

**Figure 5:**
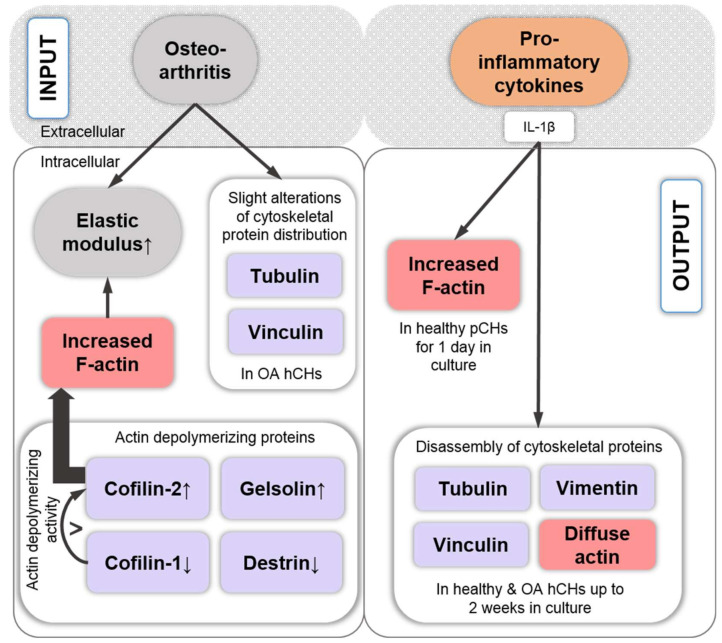
Cytoskeletal differences between healthy and osteoarthritis (OA) hCHs were detected for tubulin, vinculin, gelsolin, destrin, cofilin-1, and cofilin-2 [205]. Due to the increase in the latter and its higher actin assembly activity [206], presumably causing overall enhanced F-actin, results in elevated cell elastic moduli. The pro-inflammatory cytokine IL-1β increased F-actin in healthy pCHs that were cultured for 1 day [70], but also induced disassembly of tubulin, vimentin, vinculin, and actin in healthy and OA hCHs that were cultured for up to 2 weeks [199]. Abbreviations: IL-1β: interleukin-1β, OA: osteoarthritic, hCHs: human chondrocytes, pCHs: porcine chondrocytes. The up and down arrows indicate an increase or decrease.

Given that the SF amount and type is tightly connected to CH phenotype, this section examines cytoskeletal differences between healthy and OA CHs and argues that in OA CHs cytoskeletal changes include both actin depolymerizing and polymerizing proteins but lead to an overall actin polymerization increase. A study that non-quantitatively compared normal and OA femoral head AC hCHs identified structural differences at the nuclear, cytoplasmic, and cytoskeletal levels [199], highlighting abundant matrix fibers and secretion granules in healthy AC hCHs that were partially lost in OA AC hCHs. Additionally, β-actin localized at the apical sides of the cytosol, organized tubulin filaments from the nucleus to the periphery, and organized vimentin filaments throughout the cytoplasm were found in healthy AC hCHs, whereas OA AC hCHs displayed some alterations in tubulin and vinculin distributions but only slight changes in β-actin and vimentin distribution, compared to healthy AC hCHs. Punctated vinculin patterns were found under the plasma membrane in both healthy and OA AC hCHs. Interestingly, this group also showed that stimulation of both healthy and OA AC hCHs with the pro-inflammatory interleukin-1β (IL-1β) cytokine also led to an disassembled appearance of actin, tubulin, vimentin, and vinculin in both healthy and diseased AC hCHs [199], potentially linking the effects observed in AC hCHs with OA to IL-1β (Figure 5). This was in contrast to another study, which demonstrated that IL-1β altered the cytoskeleton by increasing the F-actin of AC pCHs, which was mediated by the RhoA pathway [70]. However, the difference in the study setup was that [199] had used isolated AC hCHs from patients for up to two weeks of culture, whereas [70] had used healthy AC pCHs and explants for overnight experiments, and therefore differences may be attributable to differences in the timeline and the species. However, both studies clearly show that IL-1β has (short- and long-term) effects on the cytoskeleton in both healthy as well as diseased OA CHs.

Another study assessed CH mechanical properties using a micropipette aspiration technique together with a viscoelastic solid cell model and demonstrated that the elastic modulus of the hip AC hCHs was significantly increased with OA, compared to healthy hCHs [200]. Using cytochalasin D for inhibiting actin polymerization decreased the AC hCH moduli up to 90% and the viscosity up to 80%, illustrating that the actin cytoskeleton contributes significantly to overall AC hCH stiffness. Interestingly, the effect of cytochalasin D was stronger in healthy than in OA hCHs but the underlying reason was not immediately apparent, as little if any differences in the cytoskeletal distributions between the two groups were observed. However, an older study had found that OA knee AC hCHs, referred to as clonal cells found in fibrillated cartilage, had varying amounts of cytoskeletal actin and vimentin, whereas healthy hCHs, referred to as non-clonal cells, had displayed relatively constant amounts of these cytoskeletal proteins [201]. In [200], subsequent exposure of femoral head AC hCHs to acrylamide, which disrupts the vimentin intermediate filament networks [202], also decreased the stiffness and viscosity in both healthy and OA CHs but with stronger effects on OA CHs, indicating that not only the actin cytoskeleton but also the vimentin network contributes significantly to overall hCH stiffness. In the same study, colchicine, used for microtubule network disruption, neither affected the moduli nor the viscosity. Together these data suggest functional differences between healthy vs. OA CHs. Interestingly, this study also demonstrated that actin, vimentin, and tubulin distributions were comparable between healthy and OA AC hCHs [200].

In this context, another study further elucidated that vimentin in knee AC hCHs formed a tight, highly interconnected inner network contained entirely within the cortical actin [203]. Fluorescence recovery experiments suggested vimentin network motion rather than individual filament turnover. Interestingly, using acrylamide for vimentin disruption revealed that a significant portion of cytoskeletal stiffness was lost when the vimentin networks were disrupted, and that cells from more arthritic cartilage were less affected. Together with references [199,200,204], these findings can be attributed to an impaired vimentin network in AC hCHs from more severely OA-affected cartilage.

Finally, a proteome analysis of knee AC hCHs derived from healthy vs. OA donors found that the expression of the actin-depolymerizing proteins destrin and cofilin-1 were downregulated in OA hCHs, whereas the expression of cofilin-2 and gelsolin, also actin-depolymerizing proteins, was upregulated in OA hCHs [205]. This is interesting because cofilin-2 has a weaker actin filament depolymerization activity than cofilin-1 and promotes F-actin assembly rather than disassembly in steady-state assays [206]. Thus, decreased cofilin-1 expression levels in OA would presumably outplay the effects of cofilin-2 and lead overall to increased actin polymerization, which, as discussed above, is associated with CH dedifferentiation. Thus, in OA, decreased cofilin-1 expression levels may contribute to OA-associated CH phenotype changes (Figure 5). Another study on OA knee AC hCHs linked increased F-actin polymerization in part to the RhoA/ROCK/LIMK/cofilin pathway [171]. Collectively, these studies highlight that the actin cytoskeleton is linked to both CH mechanical properties and phenotype. Thus, in OA, CHs cytoskeletal changes include both actin depolymerizing and polymerizing proteins and appear to lead to an overall actin polymerization increase (Figure 5).

## 8. Pro-Inflammatory Cytokine Signaling Associated with the Chondrocyte Actin Cytoskeleton

**Figure 6:**
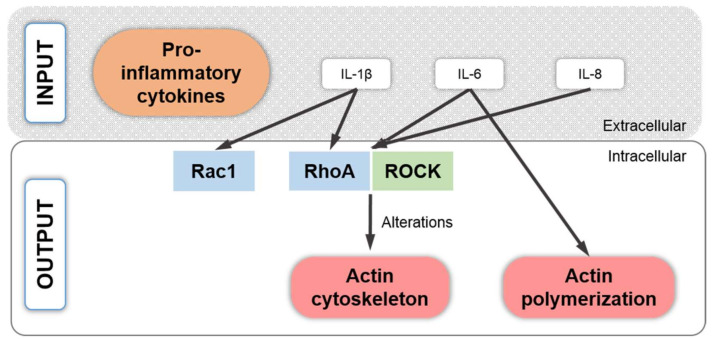
Signaling of pro-inflammatory cytokines IL-1β, IL-6, and IL-8, causing alterations in the actin cytoskeleton by signaling through the RhoA/ROCK or Rac1 pathway. Abbreviations: IL-1β: interleukin-1β, IL-6: interleukin-6, IL-8, interleukin-8, ROCK: Rho-kinase.

Changes in F-actin have been associated with the effects of the pro-inflammatory cytokines TNF-α [207,208], IL-1α [208,209], IL-1β [207,210,211], and IL-6 and IL-8 [209] (Figure 1 and Figure 6). In a study from 1997, fibronectin-coated beads coupled to articular bCHs from fetlock (ankle) joints induced the clustering of α5β1 integrin, actin accumulation, and the protein assembly of FAK, RhoA, and tyrosine-phosphorylated proteins [208]. Interestingly, treatment with a combination of IL-1α, TNF-α, and interferon gamma (IFN-γ) was shown to impair the assembly of FAK, RhoA, and F-actin, but did not disturb α5β1 integrin clustering, an early hint that pro-inflammatory cytokines impact on FA-derived signaling and SF formation. A more modern study [70] demonstrated that IL-1α increases the F-actin content of isolated adult AC pCHs cultured on glass and of CHs in situ. In another study, knee AC goat CHs (gCHs) on polyacrylamide (PAA) hydrogels coated with COL1 with different stiffnesses were treated with IL-1β [211]. As expected, CHs exposed to increased substrate stiffness displayed increased amounts of SFs, which were barely seen on 1 kPa substrates, were moderately present on 11 kPa substrates but were more prominent and parallel on 90 kPa substrates. Compared to controls, IL-1β treatment led to decreased levels of actin and vinculin staining of CHs on 1 kPa substrates, whereas IL-1β treatment did not alter the staining levels of SFs on 11 and 90 kPa COL1-coated PAA hydrogels. Other effects of IL-1β included increased cellular stiffness and lowered traction force and contracted cellular contours, suggesting that not enough strength was provided by the substrate to resist the IL-1β-induced CH contraction. Interestingly, the IL-1β effects on stiffness and traction force were more potent on soft 1 kPa substrates. Investigating the effects of IL-1β on COL2 and aggrecan synthesis, the weakest staining was in gCHs on 1 kPa substrates, indicating that the catabolic effect of IL-1β weakened with increasing substrate stiffness. These data collectively suggest pronounced IL-1β effects on cytoskeletal composition and function under permissive substrate stiffnesses.

A subsequent study demonstrated that both IL-1β and TNF-α treatment led to increased F-actin expression, cytoskeletal stiffness, and fibroblast-like cell elongation when the knee AC gCHs were cultured on plastic [207]. These data are on first glance contradictory to [211], which described a IL-1β-induced decrease in F-actin intensity but these differences can be explained by the fact that different materials with different stiffnesses (plastic vs. COL1-coated PAA hydrogels) were used and [211] demonstrated convincingly that IL-1β effects on actin are substrate stiffness-dependent with softer substrates leading to decreased F-actin levels and harder substrates to unchanged or increased F-actin levels. Another study reported a disassembled appearance of actin and also of tubulin, vimentin, and vinculin in CHs from older patients with either a femoral neck fracture or from OA patients upon stimulation with IL-1β [199], which is again in contrast to [211]. However, these differences are perhaps related to the fact that [199] used human OA or older patients’ CHs for culture on plastic for up to two weeks, whereas [211] used healthy knee gCHs for culture on COL1-coated PAA hydrogels and [207] used healthy knee gCHs on plastic for only 24 h. The effects of IL-1β on lowered traction force reported in [211] can be explained by the effects of IL-1β on multiple cytoskeletal proteins. IL-1β decreases the expression of tensin, talin, paxillin, and FAK in a dose-dependent fashion in cultured bovine AC CHs [212]. Moreover, inhibiting the paxillin–vinculin interaction (or depleting vinculin) reduces FA force transmission and depletes tugging FA traction dynamics [213], which explains how IL-1β could lower traction force.

Because IL-1β and TNF-α signal in CHs through the p38 MAPK pathway [195], it was demonstrated in [189] that stimulation of monolayer-cultured hCHs with IL-1β dose-dependently increased p38 MAPK activity within 5 h under serum-free culture conditions and led to downstream events such as matrix metalloproteinase (*MMP*)*-13* expression. Surprisingly, the addition of Y27632 led to greatly enhanced *SOX9* mRNA expression, indicating that IL-1β induces *SOX9* mRNA expression when SF formation is prevented via ROCK inhibition. The additional usage of SB202190 for inhibiting the p38 MAPK pathway decreased *SOX9* expression levels below control values, indicating that the IL-1β-induced *SOX9* expression increase was p38 MAPK-dependent but required absence of SFs. This is interesting, as it may suggest that CH *SOX9* expression in a pro-inflammatory environment induced by IL-1β can potentially be enhanced by modulating SF formation. Another interesting idea was reported in a study that used a pre-treatment of knee AC rabCHs with ibuprofen, which not only silenced the IL-1β-induced production of NO and PGE2 but also repressed the IL-1β-induced actin remodeling through the RhoA signaling pathway [210]. This can be explained by the fact that (i) IL-1β increases F-actin of CHs via RhoA [70]; and (ii) that ibuprofen can inhibit RhoA [210].

After summarizing the current knowledge on the association between the CH actin cytoskeleton and pro-inflammatory cytokine signaling, the following text briefly summarizes what is mechanistically known in this context. TNF-α appears to induce both actin depolymerization and SF formation, depending on the cell type or context investigated [214,215,216,217,218]. These varying responses can be explained by a study from 1999 [219], which demonstrated that distinct signals generated by different regions of TNF receptor 1 (TNFR-1) have specific effects on actin organization, which can mediate both a decrease and increase in F-actin. Interestingly, not only TNF-α but also granulocyte-macrophage colony-stimulating factor (GM-CSF) and granulocyte CSF (G-CSF), which human articular cartilage and CHs produce in culture in response to IL-1β [220], appear to aid actin depolymerization through the involvement of extracellular signal-regulated kinase (ERK) and/or p38 MAPK signaling (Figure 1), at least in human neutrophils [216]. Here, it was speculated that the phosphorylated actin capping protein HSP-27 is released from F-actin not only during actin polymerization but also during depolymerization [216]. In summary, IL-1β is considered to mediate its effects on the actin cytoskeleton through the small GTPases RhoA and Rac1 [70,221], whereas IL-6 induces actin polymerization [222] and both IL-6 and IL-8 are thought to signal through the Rho–ROCK pathway [223] (Figure 6). Additionally, the connection between multiple pro-inflammatory cytokines, specifically TNF-α and IL-6, with actin cytoskeleton remodeling and SF formation, might prove relevant in the context of post-traumatic AC degeneration and clinical disease because our recent evidence-based systematic review identified TNF-α and IL-6 as causal factors in inducing post-traumatic OA (PTOA) [224].

## 9. Growth Factor Signaling Associated with the Chondrocyte Actin Cytoskeleton

Growth factors, such as TGF-α, TGF-β, insulin growth factor 1 (IGF-1), and FGF-2, modulate the actin cytoskeleton, whereas bone morphogenetic protein 7 (BMP-7) regulates FAK and adaptor proteins such as tensin, talin, and paxillin. One study investigated the mechanistic aspects of the effects of TGF-α on the cytoskeleton in detail [225]. On a side note, TGF-α expression is upregulated in particular CHs in a rat model of joint destabilization-induced OA [226,227] and also in a subset of human OA samples [228]. Exposing 1-day-old Sprague–Dawley P0 knee joint rCHs cultured for 48 h on plastic to TGF-α for an additional 24 h demonstrated that GTP-bound/activated RhoA levels were increased between 4 and 30 min of treatment [225]. Subsequently, higher levels of phosphorylated MLC2 and phosphorylated LIMK1 and 2 were noted after 15 and 30 min. Additionally, phosphorylated ERK1/2 and phosphorylated Akt were elevated between 5 and 30 min after stimulation, whereas phosphorylated p38 MAPK was increased only up to 15 min, indicating the speed by which CH signaling reacts. These signaling events led to SF formation, which was prevented using the C3 Rho inhibitor or Y27632, indicating that SFs were mediated via Rho and ROCK activation, as discussed above. The effects of inhibiting TGF-α-induced mitogen-activated protein kinase kinase (MEK1/2, also known as MKK1/2 upstream of ERK)/ERK activation with the MEK1/2-specific inhibitor U0126 on SF formation was not successful. However, the TGF-α-induced downregulation of *COL2*, *SOX9*, and aggrecan mRNA expression was rescued by inhibiting MEK1/2/ERK via the MEK1/2 inhibitor, U0126. Rescue was unsuccessful via PI3K signaling inhibition using the Akt inhibitor, LY294002, via p38 MAPK inhibition (SB202190), Rho inhibition (C3), or ROCK inhibition (Y27632), highlighting that MEK1/2/ERK activation mediates chondrogenic marker expression via SOX9. Interestingly, an upregulation of *MMP-13* expression was also noted, which was not affected by inhibiting any of the pathways investigated. In contrast the inhibition of Rho/ROCK and PI3K signaling in the presence of TGF-α dramatically enhanced *MMP-13* upregulation.

TGF-α also drives pro-inflammatory cytokine expression, e.g., of *Tnfa*, which encodes TNF-α. In this context, a study [225] demonstrated that inhibiting MEK1/2 or PI3K signaling abolished basal *Tnfa* expression, whereas inhibiting MEK1/2, ROCK, p38, or PI3K signaling decreased TGF-α-induced *Tnfa* expression, illustrating the modulatory role of these signaling pathways in growth factor-induced inflammatory cytokine expression, and, in particular, the modulatory role of cytoskeleton-associated ROCK signaling in growth factor-induced inflammatory cytokine expression. Finally, the study demonstrated that TGF-α was also capable of inducing an increase in CH numbers in culture after 48 h. Moreover, inhibiting MEK1/2/ERK, Rho/ROCK, p38 MAPK, or PI3K not only prevented this increase but led to reduced CH numbers. Overall, the authors [225] concluded that TGF-α activated the epidermal growth factor receptor (EGFR) signaling, which resulted in rCHs through the activation of the RhoA/ROCK, MEK1/2/ERK, PI3K, and p38 MAPK pathways. Subsequently, TGF-α induced SFs via RhoA/ROCK signaling, whereas *COL2*, *SOX9*, and aggrecan mRNA expression were induced via MEK1/2/ERK, and, thus, *SOX9* expression rescue was possible via MEK1/2/ERK but not via Rho/ROCK inhibition.

One study that we discussed in the context of IL-1β [211] additionally investigated the effects of TGF-β1 on knee gCHs in dependence of material stiffness by using PAA gels with different stiffnesses. Compared to the controls, TGF-β1 treatment led to a pronounced increase in the actin-staining level of CHs on 90 kPa substrates but not in CHs on lower substrate stiffness, and to increased vinculin staining levels of CHs on all substrate stiffnesses but with stronger effects with increasing stiffness. Additional TGF-β1 effects led to an increased staining signal for COL2 and aggrecan and increased cellular stiffness and traction force. Interestingly, these effects were also more potent on stiffer substrates, suggesting a substrate stiffness-dependent CH response to TGF-β1 treatment that was associated with increased SF formation on higher substrate stiffness. Another group also demonstrated SF formation in TGF-β1-treated New Zealand rCHs cultured on plastic [181]. Assessing the effects of increased SF formation on cytoskeletal mechanics, one study demonstrated that TGF-β1 and IGF-1 increased the F-actin levels, which in turn were associated with increased and cytoskeletal AC bCH stiffness, supporting that CH stiffening, e.g., in response to TGF-β1 and IGF-1, is related to increased F-actin [229]. Interestingly, an older study from 1999 expanded fetal knee bCHs up to 2000-fold in the presence or absence of FGF-2 and demonstrated that the presence of FGF-2 prevented the formation of SFs that are usually associated with monolayer expansion [230].

**Figure 7:**
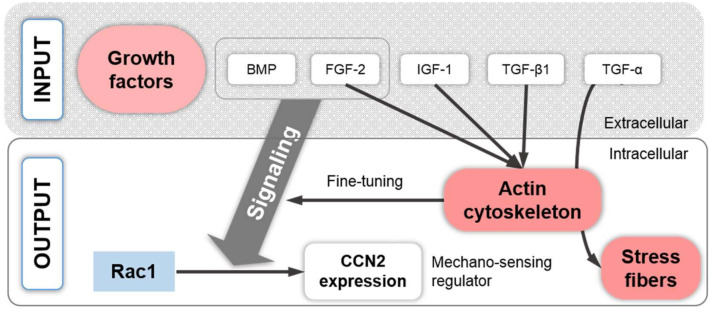
Correlation between growth factor signaling, the actin cytoskeleton, and CCN2 expression in CHs. The actin cytoskeleton modulates FGF-2 and BMP signaling through the Rac1 pathway, affecting the expression of CCN2 that functions as a mechano-sensing regulator [238]. The CH actin cytoskeleton is regulated through the signaling of the growth factors FGF-2, IGF-1, TGF-β1, and TGF-α. The latter may induce stress fiber formation. Abbreviations: BMP: bone morphogenetic protein, CCN2: connective tissue growth factor, FGF-2: fibroblast growth factor 2, IGF-1: insulin growth factor I, TGF-α: transforming growth factor α, TGF-β1: transforming growth factor β1. Arrows indicate induction.

BMP-7, which induces chondrogenesis in primary cultures of bCHs and mCHs, also increases the expression of tensin, talin, paxillin, and FAK. In turn, cytochalasin D, an actin cytoskeleton disruptor, inhibited these BMP 7-induced upregulations and also blocked the BMP-7 effects on the CH phenotype [212], indicating that BMP signaling may depend on an intact cytoskeleton. The CTGF/Cyr61/Nov (CCN) family of proteins are regulatory molecules that are generally involved in functions such as cell proliferation, angiogenesis, tumorigenesis, and wound healing [231]. Several CCN family members are affected by actin polymerization inhibition, e.g., via cytochalasin D; actin polymerization promotion, e.g., via jasplakinolide; RhoA/ROCK signaling inhibition, e.g., via Y27632; or Rac1 signaling inhibition, e.g., via NSC23766 [232]. CCN2, also known as connective tissue growth factor (CTGF), plays an essential role in articular cartilage homeostasis by regulating the proliferation and matrix degradation of CHs [233] and by fine-tuning BMP and FGF-2 signaling [234]. Interestingly, the induction of *CCN2* expression is closely related to actin polymerization in fibroblasts and osteoblasts [235], whereas in embryonic mCHs from the radius, ulna, humerus, tibia, fibula and femur, the CCN2 transcript levels are regulated by Rac1 signaling, as demonstrated by Rac1 inhibition using NSC23766, and involving somehow TGF-β/Smad signaling because the regulation of CTGF/CCN2 by Rac1 signaling is mediated via Smads [232]. The overexpression of RhoA or the inhibition of ROCK1/2 by Y27362 had no effect on *CCN2* expression [232]. In other cells types, CTGF/CCN2 is regulated by RhoA/ROCK and Cdc42 [236,237]. Additionally, in rCHs, mechanical stress-induced CCN2 also regulated the gene expression of mechanosensitive ion channels, suggesting that CCN2 acts as a mechano-sensing regulator [238]. Taken together, the CH actin cytoskeleton appears to fine-tune FGF-2 and BMP signaling via the effects of Rac1 on the expression of *CCN2* and the role of CCN2 as a mechano-sensing regulator [235]. In turn, the CH actin cytoskeleton is modulated by the signaling of growth factors such as TGF-α, TGF-β1, IGF-1, and FGF-2 (Figure 7).

## 10. TGF-β-Induced Stress Fiber Formation and Cell Stiffening

**Figure 8:**
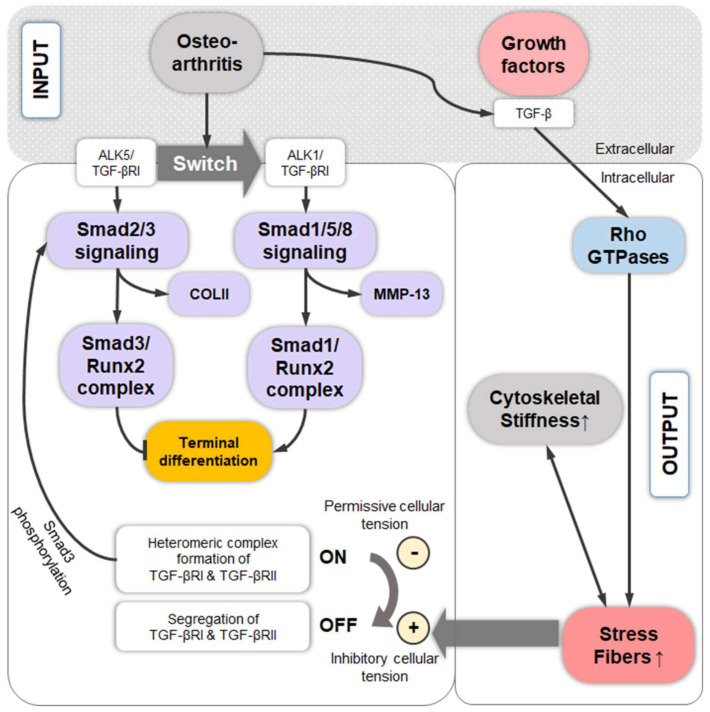
The TGF-β-induced stress fiber formation, cell stiffening, and OA-induced switch of the TGF-β receptors and downstream Smad signaling, causing terminal differentiation. During OA onset, increased TGF-β induces stress fiber formation through Rho GTPase signaling. Segregation of TGF-βRI and TGF-βRII through inhibitory cellular tension inhibits Smad3 phosphorylation, whereas a heteromeric complex formation of TGF-βRI and TGF-βRII through permissive cellular tension is required for Smad3 phosphorylation. Additionally, the receptor switches from ALK5/TGF-βRI and downstream Smad2/3 signaling, mediating COL2 production, to ALK1/TGF-βRI and downstream Smad1/5/8 signaling, mediating MMP-13 expression. Complex formation of Smad3 with Runx2 inhibits terminal differentiation, whereas the Smad1–Runx2 complex induces terminal differentiation in CHs. Abbreviations: COLII: type II collagen, MMP-13: matrix metalloproteinase 13, TGF-β: transforming growth factor β, TGF-βRI: transforming growth factor β receptor I, TGF-βRII: transforming growth factor β receptor II. The up arrow indicates an increase.

TGF-β1 plays a major role in CH phenotype regulation [239,240], in cartilage homeostasis [241], and in pro-fibrogenic processes during OA progression [242]. Altered TGF-β family signaling has been associated with aging, mechanical stress, inflammation, and OA [241,243,244]. For example, altered TGF-β signaling in ageing and OA onset has been traced back to a change in the balance of the expression of the ALK1 vs. ALK5 TGF-βRI family members [245], which has been described as a “receptor switch” from the classical ALK5/TGF-βRI-activated Smad2/3 signaling to TGF-βRI family member ALK1/ACVRL1-induced Smad1/5/8 signaling [22] (Figure 8). These findings are based on murine AC models for aging and OA and were supported by data on a range of human OA ACs, which demonstrated that the measured levels of *ALK5* and *ALK1* mRNA expression varied across samples, and that *ALK5* expression levels correlate with *COL2* levels, whereas the *ALK1* expression levels correlate with *MMP-13* expression [245]. Additionally, ALK1 knockdown results in non-detectable *MMP-13* mRNA levels in an mCH cell line (H1) from the femoral head AC [245]. Moreover, the increase in the ALK1/ALK5 ratio in OA can also explain the onset of CH hypertrophy-like changes that occur in early and late stage OA [17]. Signaling via Smad2/3 blocks CH terminal differentiation, whereas activated Smad1/5/8 signaling (ALK1) is required for CH hypertrophy [246] (Figure 8). Specifically, CH terminal differentiation is controlled by the transcription factor RUNX2, whose complex formation with Smad3 (ALK5) blocks CH terminal differentiation. In contrast, complex formation with Smad1 (ALK1) induces CH terminal differentiation [246]. In the context of this review, TGF-β-induced SF formation appears to be mediated by Rho GTPases and Smad proteins (Figure 1), as demonstrated in Swiss 3T3 fibroblasts [22,247]. In those cells, TGF-β1 and/or Smad2/3 trigger the activation of the Rho GTPase RhoA and, to a lesser extent, RhoB [247], explaining mechanistically how TGF-β signaling leads to SF formation. Interestingly, Smad3 and, to a lesser extent, Smad2 additionally induce transcription of the α-smooth muscle actin (α-SMA) gene and enhance the incorporation of α-SMA into microfilaments in Swiss 3T3 fibroblasts, increasing cytoskeletal contractility. Although such data are not available for CHs, it is interesting that approximately 75% of hCHs in the superficial zone of AC express α-SMA [248]. In this context, TGF-β1 induces SFs in both knee AC rabCHs and AC bCHs from the metatarsal joint [181,229] and in synovium-derived *rat* mesenchymal stromal cells (MSCs) [249]. Moreover, TGF-β1 increases the cellular stiffness and traction force of knee gCHs [211]. Together, these studies suggest that the cell stiffening effect of TGF-β1 is largely associated with SF formation and this appears disease-relevant, as an increase in cell stiffness was measured in femoral head AC hCHs with OA, compared to healthy hCHs [200]. Collectively, ALK5-induced Smad3/4 (ALK5) and SOX9 associate with the enhancer region of the *COL2* gene for stimulating COL2 synthesis [250]. Additionally, the Smad3 signaling blocks CH terminal differentiation, whereas Smad2/3 triggers in cells other than CHs the activation of the Rho GTPases RhoA and RhoB, potentially explaining TGF-β-induced CH SF formation and cell stiffening. In contrast, ALK1-induced Smad1 is required for CH terminal differentiation.

Another interesting study demonstrated that cellular tension regulates TGF-β receptor organization and function [251]. Specifically, a spatial segregation of the TGF-β receptors RI and RII sustains receptor inactivation, which is maintained by cellular tension. In contrast, receptor activation requires abolished spatial segregation and RI/RII complex formation, which occurs when cellular tension is disrupted. This was experimentally achieved in [251] in ATDC5 murine chondroprogenitor cells by culture on COL2-coated polydimethylsiloxane (PDMS) substrates with a relatively low stiffness of 0.5 kPa (vs. 16 kPa or vs. plastic controls) or by ROCK inhibition (Y27632, 10 µM, 15 min). Subsequent TGF-β receptor activation was demonstrated by detecting activated (phosphorylated) Smad3. Whereas these data clearly demonstrate that a specific level of “prohibitive” cellular tension maintains RI/RII segregation and TGF-β receptor inactivation, and that lowering the cellular tension activates the receptor, the effects of increased cellular tension, e.g., through SF formation, are unclear. However, the authors of the present review assume that increased tension through SF formation would maintain RI and RII segregation and sustain receptor inactivation (Figure 8). However, although the latter study provided a novel mechanism by which the cytoskeleton converts biophysical cues into a cellular response, such data are not available for CHs. Connecting the dots, these data indicate a role of specific levels of actin polymerization in regulating TGF-β receptor activation and subsequent TGF-β effects on CH function.

## 11. Cell Stiffness Is Related to the Effects of Stress Fiber Distribution vs. Amount

**Figure 9:**
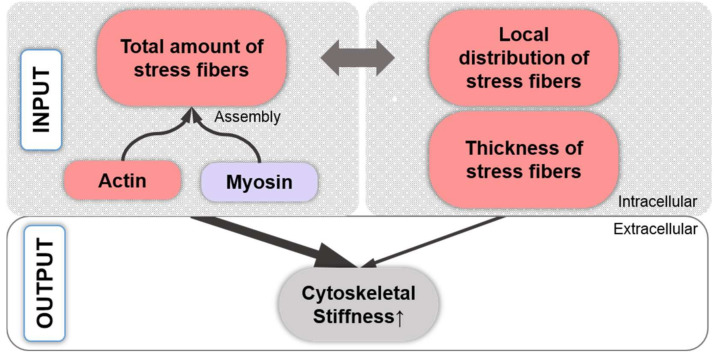
Correlation between cytoskeletal stiffness and SF amount vs. SF thickness and distribution. SF amount assembled of actin and myosin (with myosin having a higher impact) have a greater effect on global cell stiffness than SF thickness and alignment. The bold arrow indicates a larger effect than the regular arrow in the output section. The up arrow indicates an increase.

A recent study provided a mechanical model at the nanoscale that quantitatively relates physiologically relevant intracellular forces such as cytoskeletal tension and pre-stress to cell stiffness [252], illustrating the quantitative interconnectivity between cytoskeletal structures and forces. We refer the interested reader to [253] for a condensed overview on cytoskeletal building blocks, network architecture and mechanics, cytoskeletal epigenetics, and mechanosensing, which we recently have reviewed in the context of CHs and MSCs in [22]. Generally, the main determinants of cytoskeletal stiffness are the actin and myosin fiber assemblies. In adherent mouse fibroblasts (NIH3T3), an interesting study that used simultaneous atomic force microscopy and live-cell fluorescence imaging elucidated that the amount of myosin, and to a lesser extent actin, assembled in SFs directly modulated cell stiffness [254]. Moreover, the strong relationship between actin and myosin fiber amount and cellular stiffness was shown to fit well to a linear model where the spatial distribution of the SFs had a second-order modulatory effect. Accordingly, the same study determined on single-cell data that the presence of aligned and/or peripheral actin fibers resulted in cytoskeleton reinforcement and the presence of thicker myosin fibers gave rise to a higher stiffness, indicating that fiber alignment and apparent thickness have a weaker effect on global cell stiffness than fiber amount (Figure 9). Interestingly, modulating cell area had cell stiffness-modulatory effects only when cell area changes were accompanied by changes in the actomyosin fiber amount; changes in cell area without changes in actomyosin fiber amount led to a situation in which cell area and cell stiffness were decoupled. Experimentally, this situation of decoupled cell area and cell stiffness has been observed when NIH3T3 cells were allowed to spread unconstrained [254]. In contrast, cell area and cell stiffness are coupled when cells such as MSCs adhere to microcontact-printed small ECM adhesion sites for generating relatively small cell areas. In this situation, cortical stiffness was prominently affected by (small) cell area and even outpaced the effects of substrate stiffness [255]. However, perhaps the most important conclusion of [254] was that adherent cells, such as NIH3T3, can readily change their mechanical properties by either changing the total amount of SFs, which is governed by both actin and myosin assembly, or by altering the local distribution of the SFs and their thickness (Figure 9). Thus, although comparably detailed data on the intracellular mechanics of CHs is not available, comparable mechanisms likely govern CH mechanics and SF formation, disassembly, and distribution.

## 12. Regulation of TGF-β-Dependent SZP Production through the Level of Actin Polymerization

**Figure 10:**
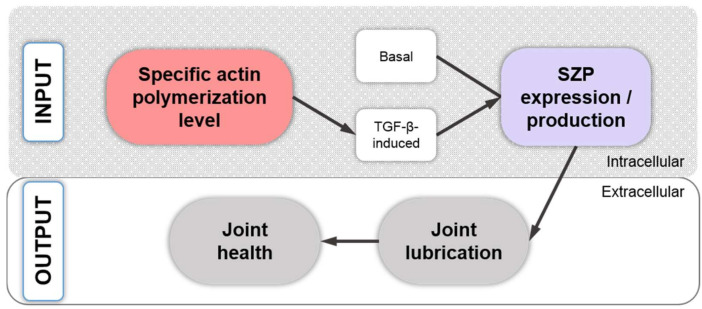
The specific range of actin polymerization causes TGF-β-induced superficial zone protein (SZP) production that results in joint lubrication and health.

A hallmark of superficial zone CHs and synoviocytes is the expression of superficial zone protein (SZP) [256,257,258], also known as lubricin of proteoglycan 4 (PRG4), which is relevant for the lubrication of articular cartilage [259]. SZP reduces the coefficient of friction and wear in articular cartilage through a sacrificial, boundary lubrication mechanism [260,261,262]. In a study that used cytochalasin D in monolayer-passaged adolescent bCHs for reversing dedifferentiation through inhibiting actin polymerization [176], cytochalasin D also modulated the expression of SZP production. Interestingly, the study differentiated between basal and TGF-β1-induced SZP production and noted that cytochalasin D treatment did not decrease basal but TGF-β1-induced SZP production. Jasplakinolide, an actin polymerizer, also dose-dependently decreased TGF-β1-induced SZP expression. These data demonstrated that the TGF-β1 response of AC bCHs from knee joints to synthesize SZP depends on a specific level of actin polymerization, as both increased the polymerization and increased depolymerization decreased TGF-β1-induced SZP production (Figure 1 and Figure 10). Because SZP serves as primary boundary lubricant in articular cartilage [263,264,265] and can hereby affect overall joint health, the actin polymerization level dependence of the TGF-β1 effects, to induce SZP production, illustrates how the CH cytoskeleton affects even joint health (Figure 10).

## 13. Fibrogenic vs. Chondrogenic Chondrocyte Expression Profiles and Catabolic Cleavage Fragment Formation Associated with the Chondrocyte Actin Cytoskeleton

A few studies have investigated the relation of the actin cytoskeleton with OA CH expression profiles, catabolic fragment generation, and *COL1* regulation [85,225,266]. One study demonstrated in P2 rabCHs isolated from the shoulder and knee joints that the long and thick actin SFs induced through passaging can be disrupted by 5 days of treatment with staurosporine [266], which is a non-selective inhibitor of protein kinases including protein kinase C (PKC) [267], and an actin filament disruptor. In line with what this review has shown so far, the treatment led to the expected induction of COL2 synthesis. Importantly, they also showed that this treatment completely inhibited COL1 as well as type III collagen (COL3) synthesis. These data indicate that COL1 and COL3 are, in addition to COL2, subject to the regulation by the actin cytoskeleton. This is because PKC is a positive regulator of chondrogenic differentiation of mesenchymal cells and, as graphically illustrated in [268], PKC, and not the inhibition of PKC, would likely inhibit *COL1* and/or *COL3* expression. In support, PKC downregulates COL1 expression in healthy human dermal fibroblasts [269] and decreases COL1 secretion in SMCs [270]. Thus, in conjunction with other text sections on COL1 and COL2 in the present review, *COL1*, *COL2*, and *COL3* expression are subject to the regulation by the actin cytoskeleton. This is relevant, as many CHs in human OA AC acquire a fibrogenic phenotype, which is characterized by upregulated *COL1* and/or *COL3* expression [242].

**Figure 11:**
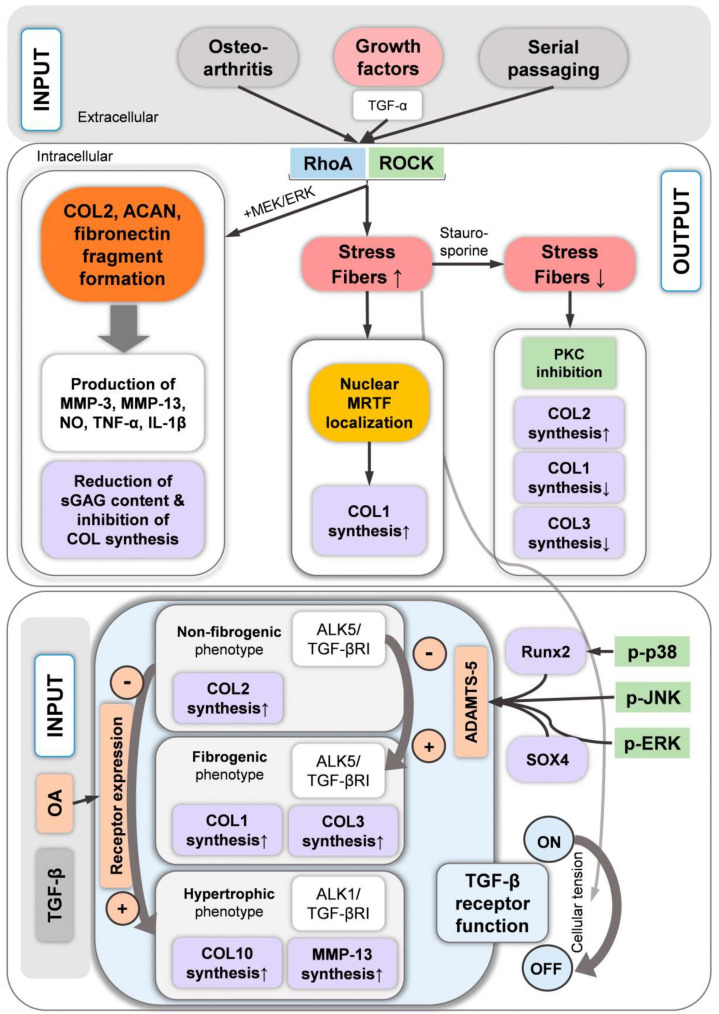
Effects of OA, growth factors, and serial passaging on stress fiber formation and the subsequent effects on TGF-β receptor function and CH phenotype. Abbreviations: ACAN: aggrecan, ADAMTS-5: a disintegrin and metalloproteinase with thrombospondin motifs 5, COL1: type I collagen, COL2: type II collagen, COL3: type II collagen, COL10: type X collagen, ERK: extracellular signal-regulated kinase, (s)GAG: (sulfated) glycosaminoglycan, JNK: JUN N-terminal kinase, MEK: mitogen-activated kinase kinase 1/2, MRTF: myocardin-related transcription factor, NO: nitric oxide, OA: osteoarthritis, PKC: protein kinase C, TGF-βRI: transforming growth factor β receptor I, TNF-α: tumor necrosis factor α, IL-1β: interleukin-1β, MMP-3: matrix metalloproteinase 3, MMP-13: matrix metalloproteinase 13, SOX4: SRY-box transcription factor 4. The up and down arrows indicate an increase or decrease.

A few studies that compared healthy vs. OA AC found upregulated *COL2* and/or *COL3* expression but not upregulated *COL10* expression in OA [271,272,273], suggesting the presence of a fibrogenic but not hypertrophic CH phenotype in OA. This is consistent with the finding that in human OA the AC is gradually replaced by fibro-cartilage [274,275,276]. Moreover, a study [242] demonstrated that a fibrogenic vs. chondrogenic response of CHs within the native AC to TGF-β1 depended on the presence vs. elimination of a disintegrin and metalloproteinase with thrombospondin motifs (ADAMTS)-5 activity. Specifically, the authors used a novel, so-called TTR model, which involves an intra-articular injection of TGF-β1 to mimic acute injury, followed by 2 weeks of uphill treadmill, to demonstrate that *ADAMTS-5* knockout mice were protected from fibrosis and AC destruction, whereas wildtype mice experienced AC destruction [277]. Subsequently, the authors reasoned in [242] that a fibrogenic phenotype is mediated by ALK5-dependent Smad2/3-signaling and represents a “soft-tissue” wound healing response, whereas terminal differentiation, as seen in OA, is mediated by ALK1-dependent signaling, representing a “fracture repair response” towards bone healing. Interestingly, the view that a fibrogenic phenotype of CHs is a downstream event of ALK5-dependent Smad2/3-signaling, and that ALK1 acts “chondrogenically” [242], does not necessarily conflict with the view of [245] that ALK1 effects are “pro-hypertrophic”. The reason is that [242] and the discussed data from [277] refer to healthy AC with a physiologically high ALK5/ALK1 expression ratio, in which TGF-β would support inhibition of hypertrophy. In contrast, a study [245] examined OA AC and demonstrated a low ALK5/ALK1 expression ratio, in which TGF-β would support induction of hypertrophy. Overall, the difference between fibrogenic, chondrogenic, or hypertrophic TGF-β signaling effects is contextual (Figure 11), based on (i) ADAMTS-5 activity; (ii) ALK5 availability relative to ALK1 availability; and (iii) the amount of available TGF-β1 because the data discussed in [242] was based on injecting TGF-β1 in a murine TTR model to mimic injury [277], whereas [245] did not use any exogenous TGF-β. However, this view would require that fibrogenic *COL1* and *COL3* expression are mediated by Smad2 and/or Smad3 signaling. Indeed, *COL1A2* and *COL3A1* as well as *COL6A1* are targets of Smad3 signaling in human dermal fibroblasts [278,279,280] and the CAGACA sequence of the *COL1A2* promoter functions as a Smad-recognition site of Smad3 [281]. Although such data are not available for CHs, these studies support the view that fibrogenic type I and III collagen expression is Smad3- and, thus, ALK5-dependent. In this complex and perhaps not entirely clear context, this review predicts a role of SF formation in regulating the fibrogenic function of ALK5, based on the above discussed study [266], which observed a modulation of *COL1*, *COL2*, and *COL3* expression by modulating SF formation, and based on [251], which reported a cellular tension-dependency of the TGF-β receptor organization and function that is discussed above. On a side note, *ADAMTS-5* regulation appears species-dependent and involves, for example, SOX4, and presumably SOX11, in OA onset in mCHs from femur and tibia AC [282], RUNX2 in SW1353 cells through phosphorylation of p38 [283], and IL-1β induces the *ADAMTS-5* and *ADAMTS-4* expression through phosphorylation of JNK and ERK1/2, but not p38 [284]. In contrast, in two studies of human knee and/or hip AC/CHs, IL-1 induced *ADAMTS-4* but not *ADAMTS-5* expression [285,286].

Another phenotype-relevant point is that [225] demonstrated that TGF-α induced COL2 and aggrecan cleavage fragment formation in cartilage explants. On a side note, COL2 fragments containing the N- and C-terminal telopeptides have dose-dependent catabolic activities similar to fibronectin fragments and increased NO, cytokine, and MMP production in AC pCHs from MCPJ [287]. To our knowledge, there is no study that has examined the relationship between fibronectin fragments and actin cytoskeleton in CHs. Moreover, in both bCHs and hCHs, COL2 fragments inhibit collagen synthesis, which is dose-dependent, e.g., in hCHs [288]. In human articular cartilage explants, COL2 fragments disturb tissue homeostasis because the fragments suppress collagen synthesis [289] and upregulate catabolic processes [288]. Importantly, TGF-α-induced COL2 and aggrecan cleavage fragment formation was greatly reduced by inhibition of MEK1/2/ERK and Rho/ROCK activation [225], suggesting that Rho/ROCK-mediated cytoskeletal changes play an active role in catabolically acting COL2 and aggrecan fragment generation. An interesting study that cultured P2 AC bCHs on plastic [85] observed that increased *COL1* expression was associated with increased levels of actin polymerization and subsequently increased nuclear localization of myocardin-related transcription factor (MRTF) (Figure 1 and Figure 11). MRTF is a G-actin binding protein, which regulates *COL1* expression in AC bCHs [85] and in lung fibroblasts [290], and whose effect on *COL1* expression can be repressed by (i) inhibiting MRTF via the small molecule CCG1423; (ii) cell rounding; or (iii) latrunculin B. Interestingly, inducing actin depolymerization in dedifferentiated AC bCHs resulted in cytoplasmic localization of MRTF and reduced *COL1* expression [85]. Overall, these few studies suggest a regulatory role of the actin cytoskeleton in (i) regulating *COL1* expression via MRTF localization; (ii) mediating a fibrogenic and catabolic CH expression profile; and (iii) modulating COL2 and aggrecan fragment generation.

## 14. Regulation of Chondrogenic *SOX9* Expression and Phosphorylation

The preceding text sections summarized the complex mechanisms that regulate actin dynamics and their effects on CH phenotype through effects on SOX9, which is a critical transcription factor for chondrogenesis and the production of cartilage-specific ECM components, such as COL2 [291]. Thus, this text section summarizes the regulation of *SOX9* expression and phosphorylation at the crossroads between actin dynamics and other relevant signaling pathways. So far, this review summarized in the context of physical stress that *SOX9* expression is induced by SAPK/p38 MAPK activity [189] but requires prevention of SF formation and low active RhoA protein levels [72,190]. In the context of induced redifferentiation of dedifferentiated rCHs, the study of [170] demonstrated that *SOX9* mRNA expression can be recovered in P4 femoral AC rCHs by using the Src inhibitor PP2, which had the stronger effect compared to the FAK inhibitor PF573228, whereas [189] demonstrated additionally that ROCK inhibition can rescue *SOX9* expression in knee AC hCHs when sufficiently long incubation times and high enough concentrations are used. In an pro-inflammatory context, IL-1β induces *SOX9* mRNA expression in P2 knee AC hCHs when SF formation is prevented via ROCK inhibition [189]. In the context of growth factors, [225] demonstrated in P0 epiphyseal rCHs from femoral condyles that TGF-α-induces EGFR signaling in CHs, which results in the activation of RhoA/ROCK, MEK1/2/ERK, PI3K, and p38 MAPK pathways. However, *SOX9* mRNA expression was induced via MEK1/2/ERK, whereas SFs were induced by RhoA/ROCK; thus, *SOX9* expression rescue was possible in this context via MEK1/2/ERK but not Rho/ROCK inhibition.

Another study [292] demonstrated in primary costal mCHs that were aged 1 to 5 days that FGF-2 at 2 ng/mL induced a 3-fold increase in *SOX9* mRNA expression and 7-fold increase in SOX9 protein expression [292]. Similarly, FGF-1 but not FGF-7 induced SOX9 protein in the CHs. Based on the fact that SOX9 targets the gene *COL2A1* for COL2 [178], the study [292] used the activity of a *COL2A1* enhancer as a functional measurement of SOX9 and demonstrated that both FGF-1 and FGF-2 increased the enhancer activity, whereas FGF-7, insulin, EGF, TGF-β, and BMP-2 did not increase the enhancer activity, indicating that selected growth factors such as FGF-1 and FGF-2, but not growth factors in general, can induce *SOX9* expression. Subsequent experiments in [292] with vectors expressing chimeric FGF receptors (FGFR) for bypassing the activation of endogenous FGFRs or wild-type FGFRs demonstrated in costal mCHs under FGF-7 stimulation that FGFR1, FGFR2, and FGFR3 can principally mediate *SOX9* expression and in C3H10T1/2 cells under FGF-2 stimulation all four FGFR1–R4 receptors can transduce signals that lead to the activation of the SOX9-dependent *COL2* enhancer element. These data are interesting because they indicate *SOX9* expression regulation by FGF-2 through all four receptors, which is important, as in human healthy and OA articular cartilage cells FGFR1 and FGFR3 dominate, compared to FGFR2 and FGFR4 [293,294]. Moreover, in human OA AC *FGFR1* expression is increased while *FGFR3* is concomitantly suppressed, compared to healthy AC [293]. However, whereas in costal mCHs the *SOX9*-inducing effects of FGF-2 are clear, the situation in human CHs appears different, as rFGF-2-mediates anti-anabolic and catabolic effects but not anabolic, COL2-related effects in human aged healthy and OA cartilage [295]. Moreover, a different study has also confirmed in knee AC hCHs and in cartilage tissue that FGF-2 overexpression enhances the survival and proliferation of both normal and OA hCHs but not *COL2* expression [296], indicating that rFGF-2 or FGF-2 overexpression in hCHs does not lead to a SOX9-mediated increase in *COL2* expression. This view is in agreement with our recent review stating that FGF-2 mediates proliferation, anti-anabolism, and catabolism in human AC [244]. Thus, it appears that FGF-2 is principally able to induce *SOX9* expression in mCHs, whereas it remains unclear whether hFGF-2 is similarly capable to induce h*SOX9*, or whether other regulatory mechanisms counteract such effects. Although glucocorticoids are not growth factors, they are also known to promote *SOX9* expression and are briefly discussed in this section. In one study, the effect of a synthetic glucocorticoid, dexamethasone (DEX), on *SOX9* gene expression in rib cage CHs of newborn mice was investigated [297]. Despite a high basal expression, DEX enhanced *SOX9* mRNA expression within 24 h and for at least up to 48 h, with dose-dependent effects starting at 0.1 nM and maximally at 10 nM. In contrast, *SOX6* expression was not affected. The treatment enhanced *COL2A1* mRNA expression and enhanced the activity of a COL2–CAT (chloramphenicol acetyltransferase) construct that contains a 1.6 kb intron fragment with a CH-specific SRY/SOX-consensus sequence, indicating *SOX9* expression induction by DEX. Finally, *COL2* expression is regulated through material stiffness through β-catenin [298] and YAP [299]. Increased material stiffness induces β-catenin nuclear accumulation through the integrin/FAK pathway [298], which stimulates β-catenin-Tcf/Lef transcriptional activity and causes decreased *COL2* and increased *COL1* expression, as demonstrated in 2-week-old New Zealand White rabCHs [300]. However, a direct interaction between α-catenin and β-catenin [300] blocks the nuclear accumulation of β-catenin and subsequently inhibits the β-catenin-induced inhibition of *COL2* expression, leading to increased *COL2* expression. On stiff substrates, an increased *YAP* expression and YAP accumulation in the nucleus has been observed to decrease *SOX9* expression in AC rCHs from humeral heads, femoral heads, and femoral condyles [299].

In the context of CH differentiation, the study of [72] found an inverse correlation between the level of activated (GTP-bound) RhoA protein but not RhoA transcript and the expression of chondrogenic transcription factors and differentiation markers in embryonal sternal cCHs. Both pan-Rho antagonists and a 3D alginate culture for inducing the redifferentiation of dedifferentiated CHs induced the expression of transcriptional regulators, such as *SOX5*, *SOX6*, and *SOX9*, together with *COL2* chondrogenic marker expression. Interestingly, the CH redifferentiation in 3D alginate culture correlated with a loss of SFs and loss of both RhoA expression and activity, indicating that chondrogenic marker expression requires low levels of RhoA protein. Using a retrovirus encoding FLAG-tagged hSOX9 (RCAS-FLAG-hSOX9), the discussed study then forced exogenous h*SOX9* expression in cCHs cultured on plastic. This led not only to increased exogenous h*SOX9* expression as expected but also to increased endogenous c*SOX9* expression, indicating the ability of SOX9 to activate its own expression and also to increase its transcriptional activity, indicated by increased chondrogenic marker expression. Repeating this experiment on RCAS-Flag-h*SOX9*-infected cCHs in a 3D alginate culture led to stable virus-encoded h*SOX9* expression and strongly increased c*SOX9* expression compared to plastic. Dissecting c*SOX9* vs. h*SOX9* expression, the strongly increased c*SOX9* expression was attributed to the effects of 3D alginate and not to h*SOX9*-induced c*SOX9* expression, as the h*SOX* expression levels remained unchanged. Moreover, these effects of 3D alginate were subsequently blocked by H89, a pharmacological inhibitor of the protein kinase A (PKA), known as adenosine 3’,5’-monophosphate (cyclic AMP)-dependent protein kinase, that also blocks phosphorylation of SOX9 at S181, as demonstrated in [72]. This was interpreted that a 3D alginate culture increases the transcriptional activity of SOX9 in a PKA-dependent fashion. On a side note, human SOX9 has three phosphorylation sites, namely, S62, S181, and S211, for PKA [301], and a study [302] demonstrated the phosphorylation by PKA at S64 and S181, whereas [72] then demonstrated that phosphorylation specifically of S181 was necessary for 3D alginate-induced actin depolymerization to enhance SOX9 function. The latter study also demonstrated that the phosphorylation site S64 or mutant phosphorylation sites, such as S181A or 181A, were not involved in 3D alginate-induced effects on SOX9 activity. Importantly, a year later, SOX9 was found to contain a phosphorylation site for ROCK and a direct ROCK–SOX9 interaction was confirmed [303]. Thus, SOX9 transcriptional activity was also linked to a ROCK-SOX9 interaction by demonstrating in an in vitro kinase assay with purified proteins that ROCK phosphorylates SOX9 at S181, which in turn increases nuclear accumulation of the SOX9 protein, e.g., in response to mechanical compression and TGF-β1 [303]. In intact cells, however, the phosphorylation of SOX9 at S181 by ROCK was only demonstrated by inducing ROCK activation through tamoxifen treatment using an estrogen reporter construct, or ROCK inhibition through treatment with hydroxyfasudil (HA-1100). Consequently, the insights reported in these two studies [72,303] connect SOX9 phosphorylation, nuclear accumulation, and activity with both PKA signaling and the effects of RhoA protein and downstream ROCK activity through a direct ROCK–SOX9 interaction. Additionally, using rat chondrosarcoma cells (RCS) and COS7 cells, the study [304] demonstrated that parathyroid hormone-related protein (PTHrP) also increased the phosphorylation of SOX9 at S181 through PKA in a dose- and time-dependent manner, whereas [305] demonstrated in that, in human CHs from healthy cartilage obtained from osteosarcoma or soft tissue sarcoma-related amputates, PTHrP induces *SOX9* mRNA expression.

**Figure 12:**
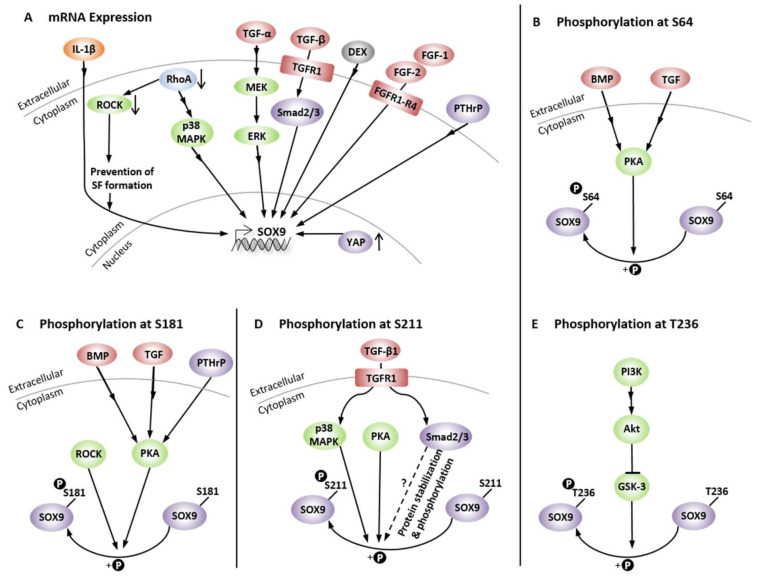
Regulation of *SOX9* mRNA expression and SOX9 phosphorylation. (**A**) mRNA expression of *SOX9* is induced through IL-1β, TGF-α or -β, FGF-1 and -2, DEX, and PTHrP in specific conditions. (**B**) Phosphorylation at S64 by PKA is induced through BMP and TGF signaling. (**C**) Phosphorylation at S181 is performed by ROCK and PKA; the latter is induced through BMP, TGF, and PTHrP signaling. (**D**) Phosphorylation at S211 is performed by PKA and p38 MAPK as well as mediated through TGF-β1 and Smad2/3 signaling. (**E**) Phosphorylation at T236 by GSK-3 is regulated through PI3K/Akt signaling. Signaling pathways and kinases involved in SOX9 phosphorylation are in part listed by [301]. Details are specified in the text. Abbreviations: BMP: bone morphogenetic protein, DEX: dexamethasone, ERK: extracellular signal-regulated kinase, FGF-1: fibroblast growth factor 1, FGF-2: fibroblast growth factor 2, GSK-3: glycogen synthase kinase 3, IL-1β: interleukin 1β, MAPK: mitogen-activated protein kinase, MEK: mitogen-activated protein kinase 1/2, PI3K: phosphatidylinositol 3-kinase, PKA: protein kinase A, PTHrP: parathyroid hormone-related protein, ROCK: Rho-kinase, SF: stress fiber, SOX9: SRY-box transcription factor 9, TGF-α: transforming growth factor α, TGF-β: transforming growth factor β, TGFR1: transforming growth factor receptor 1, YAP: Yes-associated protein.

In summary, *SOX9* expression can be induced by (i) SAPK/p38 MAPK activity under prevention of SF formation and low active RhoA protein levels, e.g., through a 3D alginate culture or C3 Rho inhibition; (ii) IL-1β; (iii) TGF-α-induced EGFR signaling via MEK1/2/ERK; (iv) DEX; (v) PTHrP; and (vi) potentially FGF-2. Moreover, YAP nuclear accumulation, e.g., induced by high material stiffness, regulates *SOX9* expression (Figure 12A). SOX9-S181 is a central site for regulating SOX9 activity, whose phosphorylation is carried out by (i) PKA; (ii) ROCK; and (iii) PTHrP through PKA. Additionally, the TGF-β-mediated SOX9 phosphorylation and stabilization in CHs is dependent on p38 activity and also Smad2/3 at S211 [306] (Figure 12C).

## 15. The Role of the Cytoskeleton in Studies Pertaining to 2D vs. 3D Culture Dimensionality, the Microenvironment, and Osmoregulation

Searching for studies that systematically compared CH SFs or the CH cytoskeleton in 2D vs. 3D conditions, in comparable microenvironments, or in osmo-adaptation studies, we noted that very few studies focused systematically on these topics. One study demonstrated that the actin cytoskeleton of AC hCHs from femoral and tibial condyles cultured in a 2D monolayer culture on plastic exhibited thick SFs that expand from one side of the cell to the other, displaying the highest intensities at the long edges [307]. In contrast, the hCHs cultured in 3D agarose (3%) hydrogels had no SFs, filopodia, or lamellipodia. Moreover, 3D cultured AC hCHs showed a cortical network of smaller woven actin filaments and punctate actin in the cytoplasm near the cell’s center. CHs in both 2D and 3D cultures had small actin projections from 1 µm to 3 µm at the cell periphery. However, the study did not account for the different cell culture substrates that were used in the 2D vs. 3D experiments. A very recent study compared OA knee AC hCHs in 2D monolayer culture on plastic with OA hCHs that were 3D-cultured within the top layer of a sponge of atelocollagen [308], which is a low-immunogenic derivative of collagen obtained by removal of N- and C-terminal telopeptide components [309]. In 3D, one group of hCHs was pre-incubated with a 1% atelocollagen solution, whereas another was not pre-incubated. Expectably, the resulting cell shape was smaller and less spread in the 3D groups than in 2D. In 2D, the metabolic activity was by approximately two orders of magnitude higher, compared to the 3D groups, whereas the highest MMP-2, -3, -9, and -13, and SOX9 and proteoglycan 4 expression levels occurred in 3D cultures with pre-incubation, compared to 3D without pre-incubation and 2D, illustrating differential effects on phenotypic marker expression. However, no COL2 data were reported. Because different cell culture substrates were used in 2D (plastic) vs. 3D (sponge ± pre-incubation) experiments, these results illustrate perhaps more microenvironmental effects than the effects of cell culture dimensionality. Indeed, the experimental groups displayed substantial variations in the expression of *ITGA2* (CD49b), the gene that encodes integrin α2. Because the α2β1 integrins bind to monomeric COL1 and are a functional cellular receptor for COL1 fibrils [310], these data illustrate microenvironmental cell adhesion-dependent effects that are superimposed on 2D vs. 3D effects. In the context of 2D vs. 3D culture effects on CH phenotype, a study that we discussed in prior text sections in more detail demonstrated that pharmaceutical actin depolymerization for reversing SF formation induced by monolayer expansion [71] led to MCPJ AC bCHs with decreased *COL1* expression and rescued the aggrecan but not *COL2* expression. 3D culture induced actin depolymerization after monolayer expansion and resulted only in decreased *COL1* mRNA expression but did not affect *COL2*, or aggrecan expression, demonstrating that actin depolymerization induced via 3D culture modulates *COL1* expression.

Expanding on this topic, an above already in detail discussed study dissected the effects of SF inhibition in 2D vs. 3D on the superinduction of *SOX9* expression via CHX [189]. Whereas *SOX9* superinduction was successful in 3D, superinduction in 2D was only successful when cytochalasin D or Y27632 were additionally used, which suggested that prevention of SF formation was required for *SOX9* superinduction in 2D. Interestingly, 3D culture without superinduction led also to the upregulation of *SOX9* expression, whereas additional pharmacological prevention of SF formation via ROCK inhibition was ineffective in 3D to increase *SOX9* expression. This was likely connected to the fact that the 3D culture already abolishes SFs, which was demonstrated in another study on dedifferentiated P5 embryonal sterna cCHs [72], and, thus, further preventing SF formation would be ineffective. However, in [72] 3D culture led in addition to SF loss also to loss of total and active RhoA protein and antagonizing RhoA via Tat-C3 transferase increased *SOX9*, *COL2*, and aggrecan expression. Thus, culture dimensionality is, of course, important for maintaining or regaining a chondrogenic CH phenotype but these studies specify collectively that 3D culture prevents SF formation and leads to low active RhoA protein. How these two points together induce or maintain chondrogenic CH marker expression is examined in more detail in the discussion section. In contrast, these studies also highlight the importance of controlling SF formation in 2D in order to control the CH phenotype, either pharmacologically or otherwise as discussed in the following text section.

A few very recent studies achieved controlling the CH morphology in a 3D culture beyond the well-known cell rounding, e.g., in alginate [189]. One study isolated MCPJ AC bCHs and used micropatterned vs. nonpatterned substrates and covered the adherent cells with a COL1 solution to generate a 3D environment termed a “a biomimetic collagenous basket” [311]. The created microenvironments were used to control bCH shape and SF formation. In accordance with that the present review has discussed so far, the study [311] demonstrated that the bCHs retained their differentiated status if the cell volume was kept constant and spreading was avoided. Another interesting study had developed micropatterned hemispheroidal wells to culture individually enclosed cells with the goal to promote a physiologically spheroidal morphology while maintaining compatibility with standard cell culture and analytical techniques [197]. The so-called “CellWells” were constructed of 15-μm-thick 5% agarose films embedded with electrospun poly(vinyl alcohol) (PVA) nanofibers. The mean diameter of 60.9 ± 24 nm of the PVA nanofibers matched the mean diameter of 53.8 ± 29 nm of human ankle COL2 fibers, whereas the nanoscale stiffness matched the published stiffness of the native pericellular matrix. Primary AC hCHs isolated from ankle joints seeded in the CellWells had maintained their spheroid morphology after 24 h more effectively than those seeded under standard conditions, illustrating the effectiveness of the introduced biphasic nanocomposite platform to control cell morphology in 3D. Thus, it is well-understood that CH morphology is an important factor that needs to be addressed for controlling the CH phenotype, which emerging developments are beginning to address.

Another topic that is connected to CH morphology and phenotype is cell volume with regard to osmo-adaptation. For example, osmolarity can affect CH proliferation and matrix production, which was shown in bCHs isolated from MCPJ AC cultured in 3D alginate beads [312]. There is increasing evidence that the actin cytoskeleton is involved in the control of cell volume and osmo-adaptation in response to hypo- and hyperosmotic stress, and that this mechanism is crucial to prevent AC and intervertebral disk degeneration [313,314]. As demonstrated by [315], which examined the response of AC bCHs to osmotic stress, sudden hypo-osmotic stress induced CH swelling followed by a regulatory volume decrease (RVD), whereas gradual hypo-osmotic stress caused only limited cell swelling without RVD. Both sudden and gradual hypo-osmotic stresses reduced the cortical F-actin intensity, whereas a sudden but not a gradual hypo-osmotic stress temporarily increased cellular CH stiffness. When using cytochalasin D, which inhibits actin polymerization, in combination with a sudden hypo-osmotic challenge, significantly lower numbers of cells exhibited RVD and the increase in cellular stiffness usually associated with RVD was abolished. These data revealed that sudden but not gradual hypo-osmotic stress activates bCH swelling, characteristic RVD, and increases stiffness, whereas gradual (and sudden) hypo-osmotic stress affects cortical F-actin intensity via actin depolymerization [315]. This is interesting because [315] linked a poor RVD to decreased actin polymerization, whereas Chapter 7 of this review summarized that CH cytoskeletal changes in OA include both actin depolymerizing and polymerizing proteins and lead to an overall increase in actin polymerization. Thus, there might be a connection between OA-induced changes in the level of actin polymerization and a change in CH RVD with OA. Indeed, OA CHs exhibit poor RVD [316]. Secondly, in a broader context, volumetric changes in CH volume affect the viability of CHs in response to mechanical injury [317] and the volume of in situ knee joint hCHs within the superficial and mid-zones is increased with AC degeneration, and to a larger extent than one would expect from increased tissue hydration in OA [318].

In other cells than CHs, cell volume regulation and osmo-sensation has been linked to the transmembrane channel “transient receptor potential vanilloid type 4 channel” (TRPV4), which has a direct molecular association with F-actin at the C-terminus [319]. TRPV4 is of functional importance in chondrocyte mechanotransduction [320], together with ion channels such as epithelial sodium channel (ENaC) and mechanosensitive Piezo channels [321], illustrating how cell volume, regulation of actin dynamics, and mechanotransduction may be linked. One study identified the expression of ENaCs in *canine* CHs (caCHs) [322] and in a prior study in hCHs. A very recent study [321] suggests that the actin cytoskeleton represents a “converging point” of various signaling pathways that modulate ENaC activity in several cell lines and the authors conclude that ENaC activity depends on the intracellular G- to F-actin balance: whereas actin disassembly resulted in ENaC activation, actin assembly led to channel inactivation.

Using HEK293 cells and mouse embryonic fibroblasts, one study [323] demonstrated that, under hyperosmotic conditions, Rac1 mediates the activity of the tonicity-responsive enhancer binding protein (TonEBP, also known as NFAT5/OREBP), an osmoregulatory transcription factor that is also expressed by CHs from AC and the intervertebral disc to maintain intracellular osmotic balance [314]. Moreover, because Arp2/3, an actin nucleator, physically interacts with “osmo-sensing scaffold for MEKK3” (OSM) [324], located downstream of TonEBP, a very recent study [314] focused on this association between cytoskeletal actin regulation and osmo-regulation and suggested that Arp2/3 plays a critical role in cartilaginous tissues such as AC and the intervertebral disc through the modulation of TonEBP-mediated osmo-adaptation. Interestingly, in that study a pharmacological inhibition of Cdc42 and Arp2/3 prevented the osmo-adaptive transcription factor TonEBP/NFAT5 from recruiting cofactors in response to a hyperosmolarity challenge, whereas mCHs with inducible *Arpc2* deletion exhibited compromised cell spreading. Mechanistically, a detailed review [325] on how the cytoskeleton regulates (decreased) intracellular volume and (increased) ionic strength in response to hyperosmotic stress accumulated convincing evidence that the small GTPases Rho, Rac, and Cdc42 are sensitive to and regulated by hyperosmotic stress. In other cells than CHs, hyperosmotic stress induces a rapid and substantial increase in the level of active Rho and, as long as hypertonicity is maintained, Rho remains activated with levels that are proportional to the applied osmotic concentration. This effect is quickly reversible upon restoration of isotonicity, which makes Rho a sensitive indicator of hyperosmotic stress and connects the regulatory mechanisms of a hyperosmotic stress response to the actin dynamics regulating processes that the present review discussed in detail in prior text sections. Moreover, hyperosmotic stress causes a fast activation of Rac and Cdc42 that is sustained during the time of hyperosmotic challenge. Their activation is accompanied by translocation to the cortical cytoskeleton, where they co-localize with cortactin, an F-actin-binding protein that promotes actin polymerization. Hypertonicity also impacts on myosin II (conventional myosin) by (i) triggering the phosphorylation of MLC; and (ii) inducing in some cells the translocation of myosin to the cell periphery. Thus, the hyperosmotic stress response appears to involve at least the small GTPases Rho/ROCK, Rac, and Cdc42, which act on both the actin and MLC components of the cytoskeleton. Collectively, these studies demonstrated the many facets of the regulatory involvement of the cytoskeleton in cell volume and the response to osmotic challenges. Recent insights demonstrate that the actin-branching Arp2/3 complex as a downstream effector of the Rho GTPases Cdc42 and Rac1 controls the osmo-adaptive transcription factor TonEBP/NFAT5 and its cofactors in response to hyperosmolarity, and that such processes influence the homeostasis of skeletal tissues. It will be exciting to see how this knowledge will be expanded to better understand how these regulatory mechanisms affect and are affected by the molecular, cytoskeletal, and phenotypical changes of the CHs in dedifferentiation and OA.

## 16. Discussion

This review summarized the existing knowledge on the intersection between mechanobiology and CH phenotype and elucidated that the cytoskeleton and the signaling processes that originate from or converge on the cytoskeleton not only impact CH function but decisively control the CH phenotype. The available knowledge suggests that CH dedifferentiation is accompanied by actin polymerization, leading to SF formation, e.g., in serial passaging or OA, and that CH redifferentiation is accompanied by actin depolymerization, leading to SF disassembly. The central question is how the causal relationship between the regulation of actin dynamics and CH phenotype looks like on the molecular level. The control of the CH phenotype assessed by the expression profile of *COL1*, *COL2*, and *COL3* is located at the interplay between (i) SF regulation and *SOX9* expression and phosphorylation, as both regulate *COL2* expression as detailed below; and (ii) MRTF cytoplasmic vs. nuclear localization, which regulates *COL1* expression.

In the context of actin dynamics and SOX9, the data reported in [72] clearly demonstrates that a chondrogenic sternal cCH phenotype requires low levels of RhoA protein. Considering that RhoA protein levels are associated with the activity of the RhoA downstream effector ROCK, low levels of RhoA protein would then speculatively be associated with accordingly low levels of ROCK. Following this train of thought, low levels of ROCK would then speculatively lead to low levels of phosphorylated SOX9 and, thus, low transcriptional activity and marker expression. So, how is it possible that low RhoA protein levels are associated with a chondrogenic phenotype when in fact the activity of the RhoA downstream effector ROCK is needed for SOX9 phosphorylation, transcriptional activity, and chondrogenic marker expression? Moreover, the study [72] demonstrated convincingly that both constitutionally activated RhoA and constitutionally activated mDia, which increase actin polymerization, inhibited the transcriptional activity of SOX9 and inhibited chondrogenic marker expression. Speculatively, would activated high RhoA levels not be associated with high ROCK activity, and would this not lead to high transcriptional activity of SOX9? In the absence of other ROCK substrates than SOX9, a ROCK-dependent increase in nuclear SOX9 in TGF-β1 treated knee AC hCHs in an alginate hydrogel and a dose-dependent induction of SOX9 transcriptional activity by ROCK have been demonstrated in vitro in SW1353 chondrosarcoma cells in a monolayer culture on plastic [303], supporting the notion that one would expect high phosphorylated SOX9 levels to be associated with high ROCK activity. How is it possible that, in fact, activated RhoA and, thus, high ROCK activity, are associated with low transcriptional activity of SOX9, as reported in [72]. This review asks the question how this apparent contradiction can be resolved.

A potential explanation could be due to ROCK substrate affinity. In addition to SOX9, at least 18 other substrates compete for a so-called ROCK consensus site that phosphorylates SOX9 and other substrates [326]. Among those substrates are the LIMKs, the ERM family proteins, MLC proteins, MLCK, and vimentin, illustrating that proteins that are associated with SF formation are also ROCK substrates. Although not much is known about the relative ROCK affinities of these substrates, various ROCK substrates can generally have a differential affinity to ROCK [327]. Thus, it is theoretically possible that, in CHs, ROCK-dependent SOX9 phosphorylation is effectively sidelined in favor of a ROCK-dependent phosphorylation of other substrates that promote SF formation, based on a higher ROCK affinity of these substrates despite high SOX9 levels. Of course, the binding of various competing substrates to ROCK might also be regulated by different levels of substrate concentration(s) and/or other regulatory mechanisms. To the best of our knowledge, no study has yet reported on this topic. However, the speculation that differential levels of ROCK substrate affinity may lead to decreased SOX9 phosphorylation in favor of SF-promoting ROCK substrates can be supported as follows. Ventral SF formation, which is a well-accepted feature of CH dedifferentiation, except when dedifferentiation is induced by IL-1 and leptin [70,171], is promoted by RhoA through its effectors, ROCK and mDia1 [75,76]. In dedifferentiating CHs, high RhoA levels, which lead to SF formation, coincide with low chondrogenic marker expression, which could speculatively be attributed to two ROCK substrates that compete with SOX9 and outpace SOX9 phosphorylation: LIMK and ezrin. For example, phosphorylation of LIMK through ROCK increases the phosphorylation and, thus, inhibition of cofilin [86], an actin-binding protein that is associated with rapid actin depolymerization, and whose inhibition leads ultimately to reduced actin depolymerization. In support of this, one study linked leptin-induced cell spreading and F-actin polymerization in knee AC hCHs indicative of induced dedifferentiation to the RhoA/ROCK/LIMK/cofilin pathway [171]. Moreover, the activation of ezrin, a member of the ERM family proteins, which modulate the cortical architecture by linking membrane-associated proteins to actin filaments at the cell cortex, supports the assembly and polymerization of cortical SFs [95,96,97,98]. Ezrin also re-activates Rho activity [328,329], building a positive feedback loop. Thus, speculatively, higher LIMK1 and/or ERM ROCK affinities than SOX9 ROCK affinity could theoretically explain increased SF formation in dedifferentiating CHs through LIMK and ERM, whereas, speculatively, a relatively low ROCK affinity of SOX9 could explain decreased chondrogenic marker expression despite high *SOX9* expression. Moreover, an increased cytoskeletal stiffness occurs in dedifferentiated AC hCHs from femoral heads in OA, compared to healthy AC hCHs [200]. The simultaneous increase in stiffness and decrease in chondrogenic markers due to CH dedifferentiation in OA can potentially be explained by a theoretical difference in the ROCK affinity of MLC vs. SOX9 because (i) MLC is a ROCK substrate whose phosphorylation correlates with increased cytoskeletal stiffness [330]; and (ii) because OA CHs exhibit decreased *SOX9* expression levels consistent with dedifferentiation [331,332,333]. Thus, the authors believe that the here-formulated, speculative theory of a differential ROCK substrate affinity of chondrogenic markers vs. SF formation-mediating substrates can explain how it is molecularly possible that dedifferentiating CHs display low chondrogenic marker expression but simultaneously high SF formation levels, although both features are RhoA–ROCK-mediated. As discussed, this theory is in accordance with CH literature on CHs. That various ROCK substrates can in general have a differential affinity to ROCK has been demonstrated in [327]. Thus, in the scenario of CH dedifferentiation, a differential affinity of competing chondrogenic vs. SF-promoting ROCK substrates may favor SF formation and disfavor SOX9 phosphorylation. In this scenario, not ROCK itself but ROCK substrate affinity and/or concentrations may constitute a master switch for CH phenotype via SF formation vs. chondrogenic expression.

**Figure 13:**
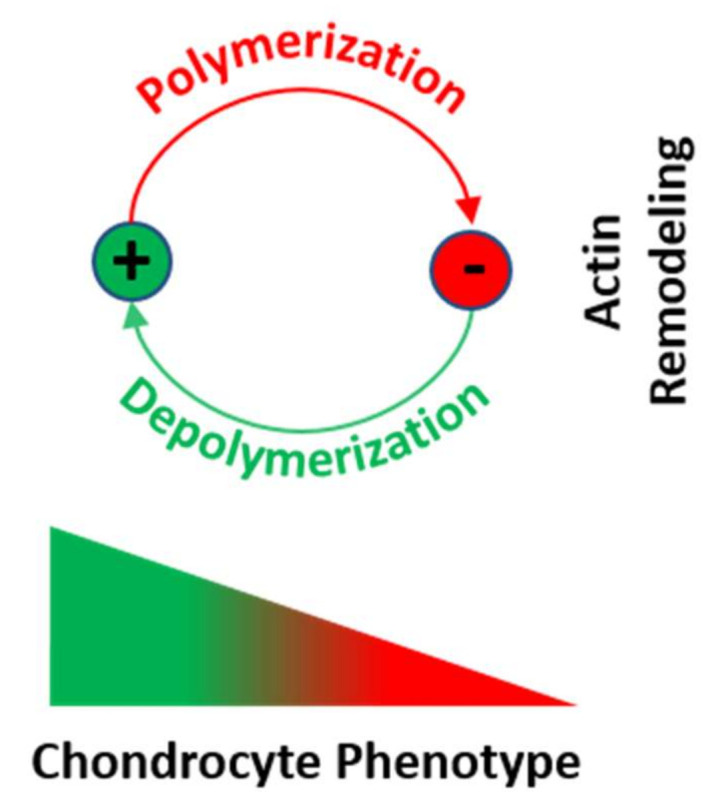
Relationship of the balance of actin polymerization and depolymerization with chondrocyte phenotype. Increased actin polymerization is associated with inducing a dedifferentiated, fibrogenic phenotype (red; indicated with by the “-“ symbol), whereas actin depolymerization is associated with regaining a chondrogenic phenotype (green; indicated with the “+” symbol).

Following this train of thought, a few studies compared P0 bCHs to serially passaged and, thus, dedifferentiated P2 AC bCHs from MCPJ [71], P4 AC bCHs from knee joints [334], and P5 sternal cCHs [72]. These studies collectively demonstrated that CH dedifferentiation is accompanied by increased actin polymerization. Subsequently, these studies attempted induced redifferentiation by reducing actin polymerization levels. For example, [71] reported that pharmacologically inducing actin depolymerization induced the redifferentiation of P2 AC bCHs from MCPJ, which led to an increased aggrecan but unchanged *COL2* expression. In [334], P4 knee AC bCH redifferentiation was achieved with cytochalasin D or staurosporine, which both induced *COL2* and *SOX9* re-expression while administered. Staurosporine additionally induced glycosaminoglycan (GAG) re-expression, which was even further augmented by adding blebbistatin, a myosin inhibitor that decreases cytoskeletal contractility. Interestingly, intermediate drug concentrations that cause a moderate degree of actin cytoskeleton re-arrangement but not a complete disruption was more productive for redifferentiation of knee AC bCHs [334], perhaps suggesting that specific levels of actin polymerization may be beneficial for a chondrogenic CH phenotype. In [72], P5 sternal cCHs were redifferentiated by administration of the ROCK inhibitor Y27632 plus co-transfection of mDia-DN, which work synergistically to depolymerize actin. This increased the induction of the *COL2* luciferase reporter by SOX9-WT by over 300%. Moreover, co-transfection of actin (R62D), a substance that blocks actin polymerization, also increased the *COL2* luciferase reporter. Collectively, these studies indicate increased *COL2* expression upon decreasing actin polymerization levels. Thus, it appears that in a healthy, differentiated CH, increasing actin polymerization levels are detrimental to a chondrogenic CH phenotype, and, vice versa, that in a dedifferentiated CH, actin depolymerization promotes a chondrogenic CH phenotype. This principle is illustrated in Figure 13.

The next question is whether the available data are in accordance with our speculative theory and do they suggest that chondrogenic vs. SF-promoting ROCK substrates compete with each other? Molecularly, a few proteins induce actin depolymerization or reduce the level of actin polymerization. For example, actin-depolymerizing factors are ADF (destrin)/cofilin and gelsolin, which both sever actin filaments. ADF and cofilin 1 (non-muscle; n-cofilin; CFL1) are able to bind to and promote steady-state F-actin disassembly to similar extents, whereas cofilin 2 (muscle; CFL2) is less efficient and also de-branches mainly “older” ADP-bound actin filaments [335]. Cofilin phosphorylation, which inhibits the interaction with actin and, thus, inhibits actin severing, is carried out by LIMK1 and LIMK2 and TES kinases (TESK1 and TESK2) [335]. Specifically, LIMK1 phosphorylation for cofilin phosphorylation and inactivation is regulated by PAK1 [336], which is a major downstream effector of the Rho-GTPases Cdc42 and Rac1 [337], and also by cAMP/PKA [338]. In contrast, LIMK2 is phosphorylated by ROCK [86,94]. Thus, although LIMK2 is a substrate for phosphorylation by ROCK, increased actin depolymerization in the context of redifferentiation of CHs would require decreased cofilin phosphorylation through decreased phosphorylation of LIMK2 through ROCK. This, in turn, illustrates that increased actin depolymerization would not impact on ROCK-mediated SOX9 phosphorylation. In contrast, these mechanisms suggest that CH redifferentiation would be accompanied by lower levels of LIMK2 phosphorylation, which may even “free-up” ROCK to phosphorylate SOX9 for increased chondrogenic marker expression. Comparable effects would be expected via the LIMK–mitogen-activated protein kinase-activated protein kinase 2 (MAPKAPK2)–p38 MAPK pathway. LIMKs are located downstream of MAPKAPK2 activity, at least in endothelial cells [107]. MAPKAPK2 activity, which is generally involved in an inflammatory response by regulating TNF and IL-6 production post-transcriptionally, is regulated through direct phosphorylation by p38 MAPK, which, in turn, is mediated by the RhoA/ROCK pathway [339]. This highlights again that ROCK activity induces cofilin phosphorylation and inactivation and subsequent inhibition of actin, suggesting that ROCK prevents actin severing via cofilin. Another actin severing protein is gelsolin. Gelsolin is inhibited by increased PIP_2_, which prevents gelsolin from severing actin. Interestingly, ROCK and also Rac and Cdc42 regulate the PIP5K isoforms, which phosphorylate (PI(4)P) to produce PIP_2_ for increasing the PIP_2_ level [136,137]. This suggests that ROCK prevents actin severing via gelsolin. In addition to the discussed actin-depolymerizing proteins, actin capping proteins, such as CapZ in muscle and adducin, bind to the barbed end and inhibit actin elongation and/or depolymerization. The CapZ regulation via RhoA/ROCK and PIP_2_ levels is comparable to that of gelsolin and suggests a ROCK-mediated actin filament stabilizing effect. In terms of adducin, ROCK phosphorylates α-adducin [340], which then binds to actin and recruits spectrin for forming a pentagonal or hexagonal scaffold [341]. This highlights again a ROCK-mediated actin filament stabilizing effect. Thus, these mechanisms suggest that the here discussed role of ROCK in mediating actin capping protein functions are related to stabilization, but not to depolymerization. Thus, increased actin depolymerization in terms of CH redifferentiation does not have an impact on ROCK-mediated SOX9 phosphorylation. If anything, actin capping protein involvement in actin depolymerization would “liberate” ROCK for increased SOX9 phosphorylation, which would subsequently increase chondrogenic marker expression. This mechanism is comparable to the above-discussed regulation of actin-depolymerizing factors. The last group of proteins involved in actin depolymerization contains actin-depolymerizing factors, such as severin and fragmin, which both sever actin filaments, as the names suggest. However, to our knowledge, there is no report that demonstrated an association of severin or fragmin with the RhoA/ROCK pathway. Thus, to summarize this text section, actin depolymerization does not involve the phosphorylation of proteins by ROCK. In contrast, the role of ROCK in the phosphorylation of proteins that are involved in actin depolymerization is related to inhibition of actin severing and inducing actin stabilization. Collectively, these data suggest that actin depolymerization as seen in CH redifferentiation does not require ROCK activity and, if anything, would “free-up” ROCK for increased levels of SOX9 phosphorylation.

The next question that arises is how SOX9 phosphorylation by other kinases than ROCK fits into the here-introduced speculative theory. More precisely, explaining CH dedifferentiation by decreased SOX9 phosphorylation due to increased ROCK-mediated SF-formation would require that CH dedifferentiation does not involve the activity of other kinases than ROCK, as active kinases would not lead to decreased SOX9 phosphorylation levels. Thus, the next text section briefly examines the role of other kinases involved in SOX9 phosphorylation. Human SOX9-S64 is subject to BMP- and TGF-signaling and is phosphorylated by PKA; SOX9-S181 is subject to BMP- and TGF-signaling and is phosphorylated by ROCK and PKA; SOX9-S211 is subject to SMAD protein signaling and, thus, to TGF-β superfamily signaling and is phosphorylated by PKA and p38 MAPK; and SOX9-T236 is subject to PI3K/Akt/mTOR signaling and is phosphorylated by GSK-3 [301]. Thus, human SOX9 has four phosphorylation sites: S181 is a ROCK target, whereas S62, S181, and S211 are PKA targets; S211 is a p38 MAPK target; and T236 is a GSK-3 target (Figure 12B–E). PKA is a cAMP-dependent protein kinase whose catalytic subunits are released in response to rising levels of cAMP and translocate to the nucleus to phosphorylate transcription factors. However, exogenous cAMP derivatives enhance chondrogenesis [268] and increased cAMP levels inhibit *MMP* expression and activity and also cartilage degradation in AC bCHs from knee joints [342]. These studies indicate that increased cAMP levels, which mediate PKA activity, were observed in a chondrogenic context and support a chondrogenesis-promoting role for PKA, as discussed in [268]. In this context, because S181 is a target of both ROCK and PKA, we point out that the experiments in [72], which reported phosphorylation of SOX9-S181 by ROCK, were based on ROCK inhibition with hydroxyfasudil (5 µM, 2 h). However, some kinase inhibitors including fasudil and hydroxyfasudil bind both kinases, ROCK and PKA. Because the Ki values of hydroxyfasudil are 0.56 µM for ROCK and 2.5 µM for PKA [343], and because the IC50 for PKA was later confirmed as low as 6 µM [344], the loss of SOX9 phosphorylation is theoretically attributable to the inhibition of PKA. However, despite these data the authors argue it is unlikely that SOX9 phosphorylation is carried out by PKA in the context of dedifferentiation because PKA activity functions in a chondrogenic context. Moreover, [345] demonstrated that PKA phosphorylates LIMK1 and enhances cofilin phosphorylation, which, as discussed above, inhibits actin severing. Similarly, although both ROCK1 and PAK1 phosphorylate LIMK1 at T508 and LIMK2 at T505 [338,346,347], the context is actin depolymerization and, thus, CH redifferentiation, as LIMK phosphorylation increases the phosphorylation and, thus, inhibition of cofilin [86], which favors reduced actin depolymerization. Consequently, in CH dedifferentiation one would not expect to observe an increased PKA activity. Therefore, in the scenario of CH dedifferentiation an increased ROCK-mediated SF-formation would not be accompanied by increased PKA activity and, thus, the low SOX9 phosphorylation levels would not be increased by PKA activity. This view is consistent with the low SOX9 phosphorylation levels that were observed in CH dedifferentiation as discussed above, which, in turn, supports our here introduced theory. The remaining kinases to be discussed are p38 MAPK and GSK-3. Reference [189] demonstrated increased SOX9 expression upon p38 MAPK signaling and reference [306] demonstrated a TGF-β-mediated phosphorylation of SOX9 at S211 through p38, highlighting the chondrogenic context of p38. This is in contrast to the context of CH dedifferentiation, in which a potential differential affinity of ROCK substrates may occur and results in low SOX9 phosphorylation levels, as observed in multiple studies. Similarly, GSK-3 induces differentiation but not dedifferentiation of cultured *murine* chondrogenic ATDC5 cells [348]. These data suggest that both p38 and GSK-3 phosphorylate SOX9 in a chondrogenic but not in a dedifferentiation context, supporting our here introduced theory. Overall, the available knowledge on this topic is in accordance with our speculative theory that CH dedifferentiation through actin polymerization is related to a differential ROCK affinity of chondrogenic (SOX9) vs. SF-promoting (LIMK2, ezrin, MLC) factors that favors SF formation and disfavors SOX9 phosphorylation, whereas CH redifferentiation through actin depolymerization promotes chondrogenic marker production through “freeing-up” ROCK for unhindered SOX9 phosphorylation. Moreover, this theory explains how it is molecularly possible that the cytoskeletal changes that occur during CH dedifferentiation concomitantly decrease a chondrogenic marker expression, and why induced CH redifferentiation, e.g., via actin depolymerization results in increased chondrogenic marker expression.

Our recent review on the effects of substrate stiffness on CH characteristics accumulated convincing evidence that multiple molecular aspects of the CH phenotype, e.g., the cytoskeletal properties, such as integrin subunit and FAK expression, SF formation, and, additionally, cell morphology, expression profiles, dedifferentiation behavior, catabolic COL2 fragment production, TGF-β1- and IL-1β-induced changes in cell stiffness and traction force, and the proliferative behavior of CHs, are susceptible to substrate stiffness [22]. The present review asked the question whether the effects of material stiffness and the above-discussed effects of passage-induced dedifferentiation on CHs share a common cytoskeleton-associated pathway. Studies show that, for example, CHs from fetal mice knee joints that were cultured on stiff (2 MPa) PDMS substrates, compared to medium and soft substrates (55 kPa, 2.1 kPa), exhibited an increase in F-actin intensity in the plasma but not cortical region after 24 h [349]. Comparable results were reported for endothelial cells [350] and mouse lung fibroblasts [159]. Generally speaking, increasing substrate rigidity induces FA protein turnover [351,352,353,354], increases the size and stability of the cell’s FAs [355,356], and increases SF assembly and adhesion [357]. The resulting increase in SFs is connected to the fact that increased matrix stiffness induces increased RhoA production and activation in the cell membrane but not in the cytosolic fraction, which subsequently increases ROCK activity [159] and mDia1, major RhoA effectors and promotors of SF formation [74,75,76]. This is supported by the observation that ROCK activity is indeed material stiffness-dependent [239]. In the context of serial passaging, multiple studies demonstrated increased SF formation with passaging-induced CH dedifferentiation [72,172,175,189]. Additionally, a study [170] demonstrated passage-dependent increases in RhoA, Rac1, and Cdc42 protein expression, together with SF thickening and increases in FA area and length, vinculin, and total FAK expression. Thus, these studies collectively demonstrate that the effects of material stiffness and passage-induced dedifferentiation are mediated by mechanisms that converge in CHs on increased FAK and RhoA activity and subsequent SF formation. In turn, the here-introduced speculative theory, reasoning that CH dedifferentiation through actin polymerization is related to a differential affinity of chondrogenic vs. SF-promoting ROCK substrates, appears applicable to both serial passaging- and substrate stiffness-induced CH dedifferentiation. Moreover, we stated in our recent review that the effects of a direct ROCK–SOX9 interaction define the CH phenotype at sub-chondrogenic and chondrogenic stiffness and that the SF-inducing effects of ROCK and subsequent induction of dedifferentiation define the CH phenotype at supra-chondrogenic stiffnesses [22]. This remains true but the present review suggests a differential ROCK affinity of chondrogenic (SOX9) vs. SF-promoting (LIMK2, ezrin, and MLC) factors that favors SF formation and disfavors SOX9 phosphorylation as a mechanistic explanation.

**Figure 14:**
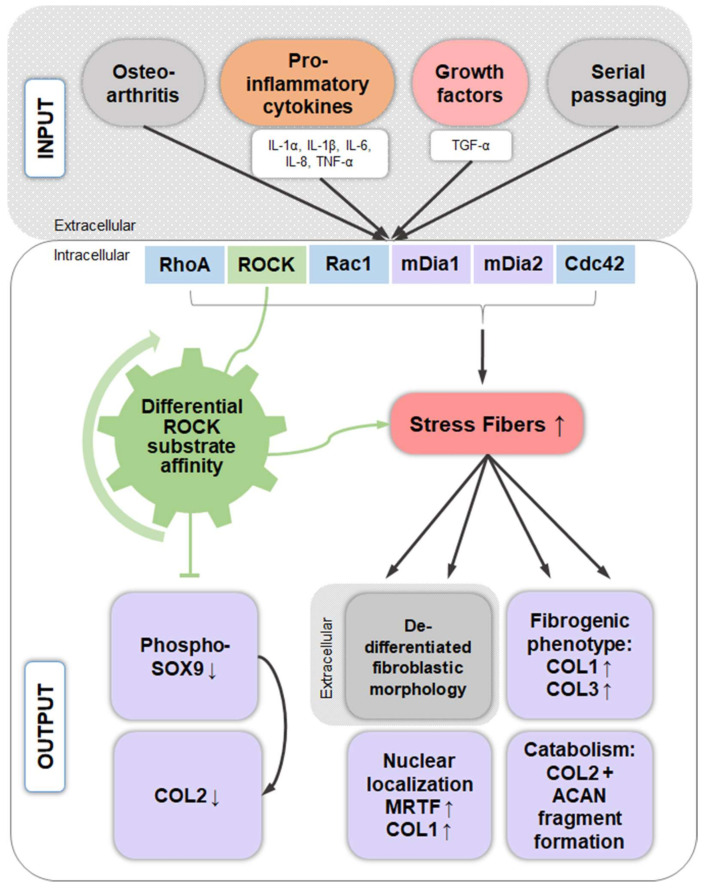
CH phenotype regulation, e.g., in OA or through serial passaging, pro-inflammatory cytokines, and/or growth factors control CH function through SF formation and the actin-regulating signaling pathways RhoA/ROCK/Rac1/mDia1/mDia2/Cdc42. SF formation in CHs induces a dedifferentiated fibroblastic morphology, a fibrogenic (increased *COL1* and *COL3* expression) and catabolic (formation of COL2 and aggrecan fragments) phenotype, and increased *COL1* expression, induced by the nuclear localization of MRTF. This review theorized that in CHs phosphorylation of SOX9 (and subsequently increased *COL2* expression as chondrogenic phenotype marker) by ROCK is effectively sidelined in favor of other SF promoting ROCK substrates (LIMK2, ezrin, and MLC), based on a differential affinity of various ROCK substrates, explaining how it is molecularly possible that dedifferentiation induces low *COL2* expression but high SF formation. Abbreviations: COL1: type I collagen, COL2: type II collagen, COL3: type III collagen, IL-1α: interleukin 1α, IL-1β: interleukin 1β, IL-6: interleukin 6, IL-8: interleukin 8, MRTF: myocardin-related transcription factor, ROCK: Rho-kinase, SOX9: SRY-box transcription factor 9, TGF-α: transforming growth factor α, TNF-α: tumor necrosis factor α. The up and down arrows indicate an increase or decrease.

This review reasoned that both serial passaging- and substrate stiffness-induced CH dedifferentiation are mediated by mechanisms that converge in CHs on increased FAK and RhoA activity and downstream SF formation (Figure 14). SF formation also alters CH morphology [170,358]. In contrast, the mechanism of how actively altering CH morphology, e.g., inducing cell rounding by using 3D culture, impacts chondrogenic marker expression is less clear. An older study noted that SF modulation described as “F-actin microfilament modification” is a sufficient signal for *COL2* re-expression and also mediates the effects of changes in cell shape and precedes any cell rounding [174]. Similarly, the authors in [189] noted that a re-differentiation-inducing knee AC hCH culture in 3D alginate, in monolayer with cytochalasin D or with ROCK inhibition, not only re-induced *SOX9* mRNA expression but was accompanied by both cell rounding and SF disruption. Thus, how do changes in CH morphology modulate SFs and CH phenotype? In a substrate stiffness-related context, the actin cytoskeleton acts like a rigidity sensor because it behaves on soft substrates like a fluid-like material and on stiffer substrates like a solid-like material, with the solid-like state characterized by a transition from an isotropic to a parallel, ordered filament organization, leading to long-lived SFs and higher tension on stiffer substrates [359]. Thus, some researchers [359] connected a polarized SF orientation with increased cytoskeletal tension, which is in general accordance with the findings in [360]. SF-induced increased cytoskeletal tension would affect the forces at individual FAs and activate FAK, as FAK localizes into FA complexes and is activated after force generation, likely through conformational changes [361]. Subsequently, the level of FAK activation would determine the activity of the downstream RhoA/ROCK pathway and regulate SFs. This assumption was based on an interesting study that determined if GEFs are responsible for RhoA activation in response to force [160]. This study demonstrated that the application of force on integrins stimulates the RhoA pathway through the GEFs GEF-H1 and LARG. However, such a mechanism does not only apply to a substrate stiffness-related context; it would also apply to a scenario in which cell morphology is actively controlled as alterations of morphology would again affect FAK activity. In detail, lower RhoA levels would (i) decrease mDia and, thus, decrease actin polymerization; (ii) decrease LIMK1 phosphorylation and, thus, decrease cofilin phosphorylation/inhibition and disassembly of the actin filaments; and (iii) decrease phosphorylation of α-adducin and, thus, decrease F-actin stabilization. Thus, it appears that controlling the CH morphology allows controlling the CH phenotype by utilizing the fact that modulating the actin cytoskeleton impacts on the FAK activity level, which subsequently controls SF formation vs. disassembly and determines the balance between insufficient vs. abundant SOX9 phosphorylation. Thus, CH shape can potentially act as both a marker of dedifferentiation [358] and as leverage point to control phenotype.

Having discussed the molecular processes that govern CH dedifferentiation vs. redifferentiation, the last questions relate to the therapeutic potential and the clinical applicability of agents that may modulate these processes. The available agents for modulating the actin cytoskeleton and the associated clinical trial phases, if any, are given in Table 2 (inhibitors of polymerization), Table 3 (stabilizers/enhancers of polymerization), and Table 4 (indirect affecters). Table 2 and Table 4 are most interesting, as CH redifferentiation or prevention of CH dedifferentiation would require prevention of actin polymerization or enhancement of actin depolymerization. Actin inhibitors (Table 2), such as chaetoglobosin A and J [362,363], cytochalasin B and D [364,365], latrunculin A and B [366,367], and urolithin A, which was only recently shown to decrease actin polymerization [368], have already been evaluated in clinical trials. Some ROCK inhibitors (Table 4) have also been tested in all clinical trial phases for the treatment of glaucoma and ocular hypertension [369] and could play a role in the treatment of acute lung injury [370]. Three ROCK inhibitors that indirectly induce F-actin depolymerization through blocking the signaling pathway that leads to NMMII activity [371] are approved for clinical use: fasudil in Japan for the treatment of cerebral vasospasm (1995) [372]; ripasudil, also in Japan, for treatment of glaucoma (2014) [372,373,374]; and netarsudil, which was authorized in the US after FDA approval [375]. Fluvoxamine, which is a selective serotonin uptake inhibitor that inhibits actin polymerization, is approved as an anti-depressant [376]. Moreover, the ARPC2 inhibitor pimozide [377], the tyrosine kinase inhibitor dasatinib [378], and the phosphodiesterase inhibitor papaverine [379] have been tested in several clinical trial phases. Overall, several agents are being evaluated, have been evaluated, or are even licensed for a variety of diseases but none is cartilage-related and further data will be required to judge their potential in a cartilage-related context, such as modulating the balance between actin polymerization and depolymerization to generate a chondrogenic phenotype.

In summary, this review elucidated that multiple CH phenotype regulators control CH function through the cytoskeleton and cytoskeleton-regulating signaling processes (Figure 14). Specifically, serial passaging, pro-inflammatory cytokine signaling (TNF-α, IL-1α, IL-1β, IL-6, IL-8), growth factors (TGF-α), and OA not only induce dedifferentiation but also converge on RhoA/ROCK/Rac1/mDia1/mDia2/Cdc42 to promote SF formation. These affect the CH morphology and subsequently the CH phenotype, potentially via a differential affinity of ROCK substrates that disfavors SOX9 phosphorylation and chondrogenic marker expression. Moreover, the CH actin cytoskeleton regulates *COL1* expression, modulates COL2/aggrecan fragment generation, and mediates a fibrogenic/catabolic expression profile, demonstrating that actin dynamics-regulating processes decisively control the CH phenotype. In contrast, modulating the CH morphology effectively allows modulating the FAK and RhoA activity and SF presence by modulating the levels of actin polymerization/depolymerization. This, in turn, can lower the LIMK phosphorylation levels and may “free up” ROCK to phosphorylate SOX9 for increased chondrogenic marker expression. Collectively, the present review revealed that numerous molecular signaling pathways converge with the CH actin cytoskeleton on the CH phenotype and introduced a speculative theory that explains molecularly how SF formation induces loss of the CH phenotype.

## Figures and Tables

**Table 1:** Definition of terms regarding the actin cytoskeleton.

Term	Definition	Ref.
Actin nucleation	“Initial assembly of G-actin monomers to form multimers”	[37]
Actin polymerization	“Addition of G-actin monomers for the formation of polymerized filaments”	[37]
F-actin	“Polymerized actin filaments consisting of two helically coiled chains”	[38]
SFs	“Multiple actin filaments crosslinked by α-actinin and/or NMMII and/or other proteins”	[41,42,44]

**Table 2:** The effects of inhibitors on the actin cytoskeleton and information on tests in clinical trials. The cited references describe the effect on actin; information on the clinical trials was collected from clinicaltrials.gov. N/A: not applicable.

Drug Treatment (Inhibitors)	Effect	Ref.	Clinical Trial Phase	Disease	Studies
Aplyronine A	Sequesters G-actin, inhibits polymerization, and depolymerizes F-actin	[380]	-	-	0
Bistramide A	Represses polymerization and depolymerizes F-actin	[381]	-	-	0
Chaetoglobosin A	Targets F-actin and inhibits actin polymerization	[362]	N/A	Infertility	1
Chaetoglobosin J	Depolymerizes F-actin	[363]	-	-	0
Cytochalasin B	Binds to protomers at the actin filament and disrupts them	[364,382]	N/A	Infertility	2
Cytochalasin D	Caps barbed ends, disrupts actin filaments, binds to G-actin and inhibits interaction with cofilin	[364,365]	N/A	Infertility	1
Halichondra- mide	Caps barbed ends and severs F-actin	[366]	-	-	0
Kabiramide A	Binds highly specific to F-actin	[383]	-	-	0
Latrunculin A	Forms complex with G-actin and thus prevents polymerization and induces F-actin polymerization	[366]	N/A	Infertility	3
Latrunculin B	Forms complex with G-actin and thus prevents polymerization and induces F-actin polymerization (diminished effect compared to latrunculin A)	[366,367]	N/A	Infertility	1
Lobophorolide	Inhibits filament growth probably through barbed end capping	[384,385]	-	-	0
Misakinolide A (Bistheonellide A)	Sequesters G-actin, inhibits polymerization, depolymerizes F-actin, caps but does not sever F-actin	[386,387]	-	-	0
Mycalolide B	Quickly depolymerizes F-actin	[383,388]	-	-	0
Pectenotoxin 2	Sequesters G-actin	[366]	-	-	0
Peroxynitrite	Causes F-actin depolymerization	[389]	-	-	0
Scytophycin C	Inhibits polymerization and induces F-actin depolymerization	[390,391]	-	-	0
Sphinxolide / Reidispongiolide	Inhibits actin polymerization	[390,392]	-	-	0
Swinholide A	Severs F-actin	[386]	-	-	0
Tolytoxin	Inhibits actin polymerization and induces depolymerization	[393,394]	-	-	0
Ulapualide A	Depolymerizes actin	[390,395]	-	-	0
Urolithin A	Decreases actin polymerization	[368]	N/A	“Healthy aging”	1

**Table 3:** The effects of stabilizer or enhancer on the actin cytoskeleton and information on tests in clinical trials. The cited references describe the effect on actin; information on clinical trials was collected from clinicaltrials.gov.

Drug Treatment (Stabilizer / Enhancer)	Effect	Ref.	Clinical Trial Phase	Disease	Studies
Bisebromoamide	Enhances G-actin polymerization and inhibits F-actin depolymerization	[394]	-	-	0
Amphidinolide H	Diminishes actin depolymerization	[396]	-	-	0
Chondramide C	Induce G-actin polymerization	[397]	-	-	0
Doliculide	Stabilizes and overpolymerizes actin filaments	[398]	-	-	0
Jasplakinolide (jaspamide)	Causes rapid nucleation of actin polymerization, stabilizes actin filaments	[399]	-	-	0
Lithium	Increases actin nucleation, enhances actin polymerization	[400]	Early-4	Several	439
Lyngbyabellin C	Stabilizes actin filaments	[394]	-	-	0
Miuraenamide A	Enhances G-actin polymerization and inhibits F-actin depolymerization	[394]	-	-	0
Phalloidin	Stabilizes actin oligomers	[366]	-	-	0
Seragamide A	Stabilizes actin filaments	[401]	-	-	0

**Table 4:** The effects of drugs that indirectly affect the actin cytoskeleton and information on tests in clinical trials. The cited references describe the effect on actin; information on clinical trials was collected from clinicaltrials.gov.

Indirect Actin Affecters	Inhibitor of	Effect	Ref.	Clinical Trial Phase	Disease	Studies
Benpro- perine phosphate	ARPC2	Inhibits actin polymerization	[402]	-	-	0
Pimozide		Delays the ARP2/3-mediated actin polymerization	[377]	2–4	Several	13
RA306	CaMKII	Bundles actin filaments	[403]	-	-	0
Blebbistatin	NMMIIb	Blocks the ATPase activity of NMMII and thus, depolymerizes actin	[371]	-	-	0
Papaverine	Phospho- diesterase	Causes F-actin depolymerization	[379]	Early-4	Several	22
AR-12286	ROCK	Block the signaling pathway that induces NMMII activity and thus, depolymerize actin stress fibers [371]	[404,405,406]	1, 2	Glaucoma, Ocular hyper- tension	12
Fasudil ^1^ (HA-1077)			[407,408]	2–4	Several	10
H-1152			[409]	-	-	0
INS117548			[410]	1	Glaucoma	1
Netarsudil ^2^ (AR-13503)			[375,411,412,413,414,415]	Early-4	Several	24
Ripasudil ^3^ (K-115)			[373,374,416,417]	2, 4	Fuchs’ Endothelial Dystrophy	3
SNJ-1656			[418]	yes (1, stated in [418])	Glaucoma	1
Y27632			[371]	-	-	0
Fluvo- xamine ^4^	Selective serotonin uptake	Inhibits actin polymerization	[376]	1–4	Several	66
Dasatinib	Tyrosine kinase	Inhibits F-actin reorganization	[378]	Early-4	Cancer, mainly leukemia	324

^1^ Fasudil is approved for treatment of cerebral vasospasm in Japan [372].^2^ Netarsudil is approved by the FDA for treatment of glaucoma or ocular hypertension in the US [375].^3^ Ripasudil is approved for treatment of glaucoma or ocular hypertension in Japan [374].^4^ Fluvoxamine is approved for clinical use as an anti-depressant [376].

## Abbreviations

2D	Two-dimensional
3D	Three-dimensional
α-SMA	Alpha smooth muscle actin
AC	Articular cartilage
ACVRL1/ALK1	Activin A receptor type II-like kinase 1
ADAMTS	A disintegrin and metalloproteinase with thrombospondin motifs
ADF	Actin-depolymerizing factor
AIP1	Actin-interacting protein 1
ALK5	Activin receptor-like kinase 5
Arp2/3	Actin-related protein complex 2/3
bCHs	Bovine chondrocytes
BMP	Bone morphogenetic protein
CAP	Cyclase-associated protein
CAT	Chloramphenicol acetyltransferase
CBP	CREB-binding protein
cCHs	Chicken chondrocytes
caCHs	Canine chondrocytes
CCN2/CTGF	Connective tissue growth factor
CECs	Corneal endothelial cells
CH(s)	Chondrocyte(s)
CHX	Cycloheximide
CIN	Chronophin
COL1/2/3/10	Type I/ II/ III/ X collagen
cSOX9	Chicken SRY-box transcription factor 9
DEX	Dexamethasone
DRF1	Dehydration responsive factor 1
ECM	Extracellular matrix
EGF	Epidermal growth factor
EGFR	Epidermal growth factor receptor
ENaC	Epithelial sodium channel
ERK	Extracellular signal-regulated kinase
ERM	Ezrin/radixin/moesin
F-actin	Filamentous actin
FAK	Focal adhesion kinase
FAs	Focal adhesions
FGF-1 / 2	Fibroblast growth factor 1 / 2
FGFR1 / 2 / 3 / 4	Fibroblast growth factor receptor 1 / 2 / 3 / 4
G-actin	Globular actin
GAG	Glycosaminoglycan
GAP	GTPase-activating protein
gCHs	Goat chondrocytes
G-CSF	Granulocyte colony-stimulating factor
GEF	Guanine-nucleotide exchange factor
GM-CSF	Granulocyte-macrophage colony-stimulating factor
GRAF	GTPase regulator associated with FAK
GSK-3	Glycogen synthase kinase 3
hCHs	Human chondrocytes
hSOX9	Human SRY-box transcription factor 9
HSP-27	Heat shock protein 27
IFN-γ	Interferon gamma
IGF-1	Insulin growth factor 1
IL-1(α/β)	Interleukin 1 (alpha/beta)
IL-6	Interleukin 6
IL-8	Interleukin 8
JNK	JUN N-terminal kinase
LIMK	LIM domain kinase
MAL	Megakaryoblastic leukemia 1
MAPK	Mitogen-activated protein kinase
MAPKAPK2	Mitogen-activated protein kinase-activated protein kinase 2
mCHs	Murine chondrocytes
MCPJ	Metacarpophalangeal joints
mDia1/DIAPH1/DRF1	Mammalian Diaphanous 1
MEK1/2	Mitogen-activated protein kinase / ERK kinase
MKK	Mitogen-activated protein kinase kinase
MLC	Myosin light chain
MLCK	Myosin light chain kinase
MLCP	Myosin light chain phosphatase
MMP	Matrix metalloproteinase
MPs	Micropattern
MRCKα	Myotonic dystrophy-related Cdc42-binding kinase alpha
mRNA	Messenger ribonucleic acid
MRTF	Myocardin-related transcription factor
MSCs	Mesenchymal stem cells
NMMII(A/B)	Non-muscle myosin II A/B
NO	Nitric oxide
NPFs	Nucleation-promoting factors
OA	Osteoarthritis / Osteoarthritic
OSM	Osmo-sensing scaffold for MEKK3
P	Passage
Pa	Pascal
PAA	Polyacrylamide
PAK	p21-activated kinase
pCHs	Pig / porcine chondrocytes
PDMS	Polydimethylsiloxane
PGE2	Prostaglandin E2
PI3K	Phosphatidylinositol 3-kinase
PI4P	Phosphatidylinositol-4-phosphate
PIP_2_	Phosphatidylinositol (4,5)-bisphosphate
PIP_3_	Phosphatidylinositol (3,4,5)-trisphosphate
PIP5K	Phosphatidylinositol 5-kinase
PKA	Protein kinase A
PKC	Protein kinase C
PKD	Protein kinase D
PLCγ	Phospholipase C gamma
PP1	Protein phosphatase type 1
PP2	4-amino-5-(4-chlorophenyl)-7-(dimethylethyl)pyrazolo[3,4-*d*]pyrimidine
PP2A	Protein phosphatase type 2A
PP2B	Protein phosphatase type 2B
PRG4	Proteoglycan 4
PSGAP	PH- and SH3-domain-containing RhoGAP protein
PTHrP	Parathyroid hormone-related protein
PTOA	Post-traumatic osteoarthritis
PVA	Poly(vinyl alcohol)
rabCHs	Rabbit chondrocytes
rCHs	Rat chondrocytes
RCS	Rat chondrosarcoma
ROCK	Rho-associated kinase
RVD	Regulatory volume decrease
SAPK	Stress-activated protein kinase
SF	Stress fiber
siRNA	Small interfering ribonucleic acid
SMC	Smooth muscle cell
SOX4 / 5 / 6 / 9	SRY-box transcription factor 4 / 5 / 6 / 9
SRF	Serum response factor
SSH	Slingshot
SZP	Superficial zone protein
TESK1 / 2	Testicular protein kinase 1 / 2
TGF-α	Transforming growth factor alpha
TGF-β(1)	Transforming growth factor beta (1)
TNF-α	Tumor necrosis factor alpha
TNFR-1	Tumor necrosis factor receptor 1
TonEBP	Tonicity-responsive enhancer binding protein
TRPV4	Vanilloid type 4 channel
VASP	Vasodilator-stimulated phosphoprotein
VEGF	Vascular endothelial growth factor
YAP	Yes-associated protein
